# Supplementary material for: Archaeometallurgical Analysis of the Provincial Silver Coinage of Judah: More on the Chaîne Opératoire of the Minting Process
Source: Materials (Basel). 2023 Mar 9;16(6):2200. doi: 10.3390/ma16062200 (PMC10052650; doi:10.3390/ma16062200)
Supplement: Supplementary file 1 [file materials-16-02200-s001.zip › materials-2248739-supplementary.pdf]

Electronic Supplementary Material – Materials, MDPI

Archaeometallurgical Analysis of the Provincial Silver Coinage of Judah: More on the *Chaîne Opératoire* of the Minting Process

Maayan Cohen [1,2], Dana Ashkenazi [3,*], Haim Gitler [4] and Oren Tal [1]

1 Department of Archaeology and Ancient Near Eastern Cultures, Tel Aviv University, Ramat Aviv, Tel Aviv 6997801, Israel
2 Leon Recanati Institute for Maritime Studies, University of Haifa, Haifa 3498838, Israel
3 School of Mechanical Engineering, Tel Aviv University, Ramat Aviv, Tel Aviv 6997801, Israel
4 Israel Museum, Derech Rupin 11, Jerusalem 9171002, Israel
* Correspondence: danaa@tauex.tau.ac.il or dana@eng.tau.ac.il

In this Electronic Supplementary Material file SEM images and SEM-EDS analysis results (Figures S1–S8 and Tables S1–S9) of die-linked Persian period Yehud *gerah* silver coins Type 5 (O1/R1, O1/R2, O1/R3, O1/R4, O1/R5), Yehud half *gerah* coins Type 16 O2/R2, and Macedonian period Yehud quarter *obol* coins Type 24 O1/R2, and Yehud *hemiobol* coins Type 31 O1/R1 are presented. The current research included SEM-EDS analyses of silver coins (320 measurements). An example

e of SEM-EDS spectra of a typical coin's surface is shown in Figure S1. The photographs of all the coins are presented in Figure S9–S16.

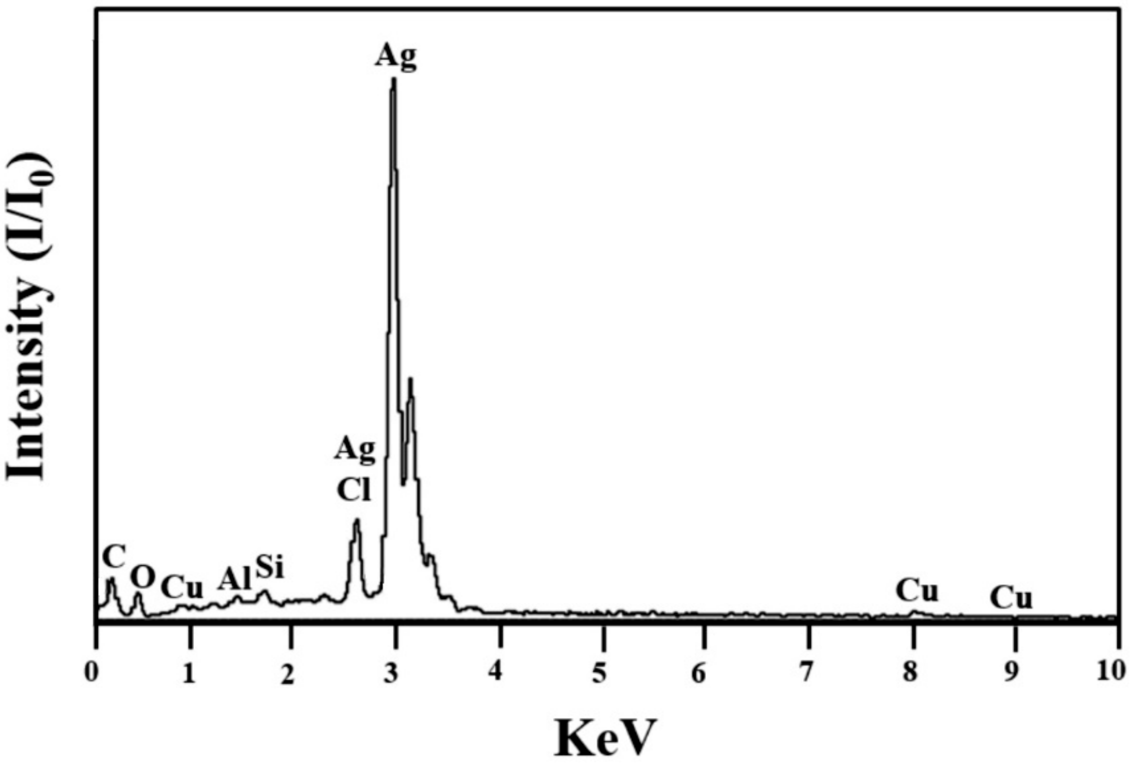

Figure S1. SEM-EDS analysis showing the spectra of a typical Yehud *gerah* silver coin (Type 5 O1/R3, IAA 153978, reverse).

1. Yehud *Gerah* Type 5 (O1/R1, O1/R2, O1/R3, O1/R4, O1/R5)

Eight Type 5 O1/R1 coins were examined in the current study: IAA 138139, IAA 153975, IMJ 27424, IMJ 27398, IMJ 34542, IMJ 34539, IMJ 34543, and IAA 153976. The SEM images of the Yehud *gerah* coins Type 5 O1/R1 (obverse with helmeted head of Athena and reverse with owl and a Paleo-Hebrew inscription) shows the bright silver metal regions according to BSE mode (inside the squares) that were examined by EDS analysis (Figure S2). The SEM-EDS analysis results of eight Yehud *gerah* Type 5 O1/R1 specimens' surfaces (obverse and reverse) are presented in Table S1. The composition of coin no. IAA 153976 was different than the composition of the other seven coins (Table S1), and therefore was not included in the average value and standard deviation (SD) calculations of the Type 5 O1/R1 coins' alloy composition (main article).

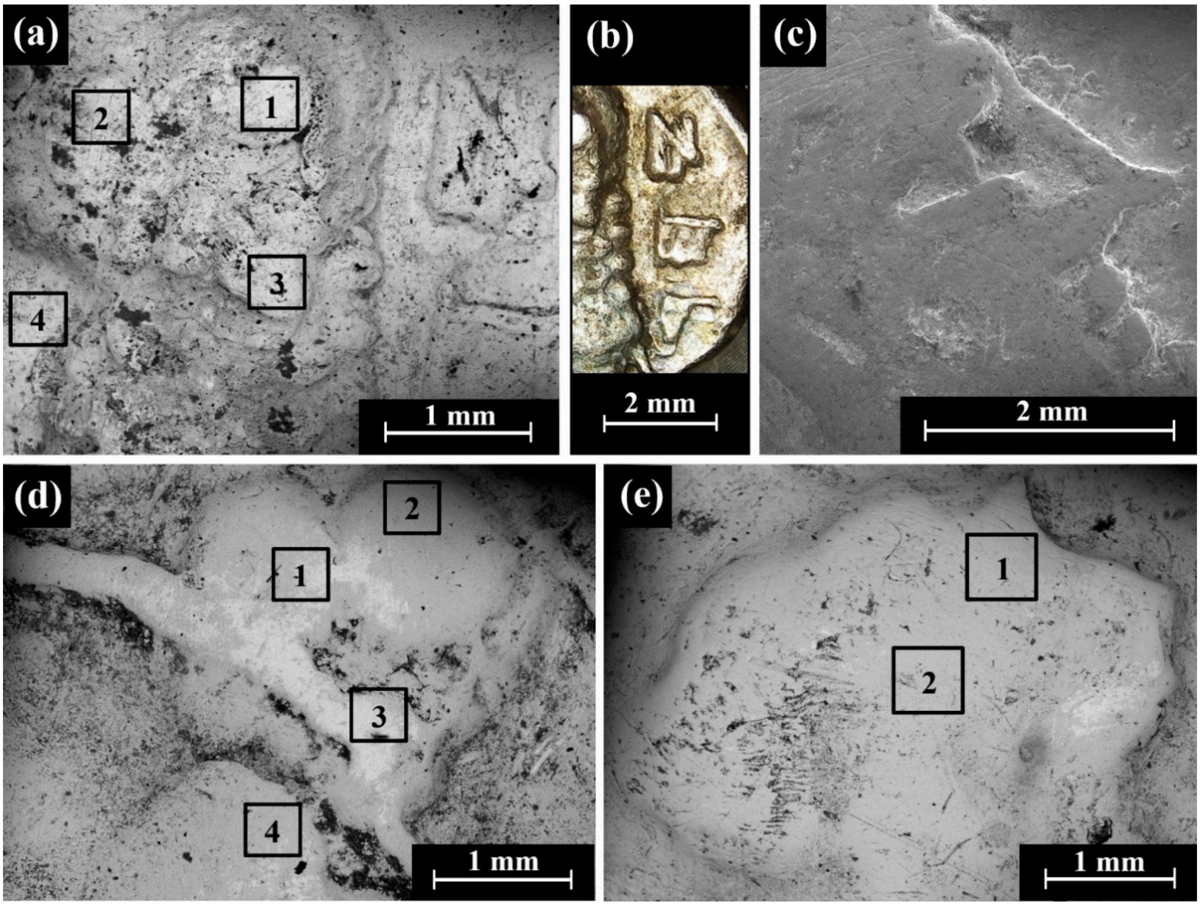

Figure S2. Images of the Yehud *gerah* Type 5 O1/R1 coins: (a) IAA 138139 reverse depicting an owl (SEM, BSE mode); (b) IAA 138139, reverse (multi-focal LM) showing the Paleo-Hebrew inscription YHD (Yeh[u]d); (c) IMJ 27398 obverse depicting a helmeted Athena (SEM, SE mode); (d) IMJ 34543 reverse depicting an owl (SEM, BSE mode); and (e) IMJ 34543 obverse depicting a helmeted Athena (SEM, BSE mode). The areas inside the squares were examined by EDS analysis.

Table S1. SEM-EDS analysis results of the Yehud *gerah* Type 5 O1/R1 coins, where SA represents the scanned area. Only bright areas according to BSE mode of shiny metal were examined by EDS analysis.

**Sample**	**Composition (wt %)**							
	**Surface**						**Silver alloy**	
	**Ag**	**Cu**	**O**	**Si**	**Cl**	**Others**	**Ag**	**Cu**
IAA 138139, reverse, area 1 (Figure S2a), SA: 300 μm × 300 μm	82.1	3.2	12.6	0.9	1.2	–	96.2	3.8
IAA 138139, reverse, area 2 (Figure S2a), SA: 300 μm × 300 μm	76.2	5.6	15.8	1.5	0.9	–	93.2	6.8

IAA 138139, reverse, area 3 (Figure S2a), SA: 300 µm × 300 µm	79.0	5.1	13.2	2.1	0.6	–	93.9	6.1
IAA 138139, reverse, area 4 (Figure S2a), SA: 300 µm × 300 µm	76.3	4.9	17.3	0.9	0.6	–	94.0	6.0
IAA 138139, obverse, area 1, SA: 800 µm × 800 µm	75.0	4.0	18.0	1.5	0.9	0.6 Ca	94.9	5.1
IAA 138139, obverse, area 2, SA: 800 µm × 800 µm	84.4	1.9	12.1	0.9	0.4	0.3 S	97.8	2.2
IAA 153975, reverse, area 1, SA: 300 µm × 300 µm	90.1	1.0	4.2	–	–	4.7 Sn	98.9	1.1
IAA 153975, reverse, area 2, SA: 300 µm × 300 µm	90.9	2.0	7.1	–	–	–	97.8	2.2
IAA 153975, reverse, area 3, SA: 300 µm × 300 µm	93.4	4.1	2.5	–	–	–	95.8	4.2
IAA 153975, reverse, area 4, SA: 300 µm × 300 µm	88.8	3.0	7.4	0.8	–	–	96.7	3.3
IAA 153975, obverse, area 1, SA: 800 µm × 800 µm	89.0	4.4	5.7	0.4	–	0.5 Ca	95.3	4.7
IAA 153975, obverse, area 2, SA: 800 µm × 800 µm	89.1	4.0	6.5	0.4	–	–	95.7	4.3
IMJ 27424, reverse, area 1, SA: 300 µm × 300 µm	94.7	–	4.6	0.7	–	–	100	–
IMJ 27424, reverse, area 2, SA: 300 µm × 300 µm	95.6	–	4.4	–	–	–	100	–
IMJ 27424, reverse, area 3, SA: 300 µm × 300 µm	95.8	–	3.8	0.4	–	–	100	–
IMJ 27424, reverse, area 4, SA: 300 µm × 300 µm	87.2	–	9.7	0.8	1.4	0.9 S	100	–
IMJ 27424, obverse, area 1, SA: 800 µm × 800 µm	89.1	–	9.1	0.8	1.0	–	100	–
IMJ 27424, obverse, area 2, SA: 800 µm × 800 µm	97.6	–	–	0.9	1.5	–	100	–
IMJ 27398, reverse, area 1, SA: 300 µm × 300 µm	68.7	2.1	8.8	1.8	17.9	0.7 Al	97.0	3.0
IMJ 27398, reverse, area 2, SA: 300 µm × 300 µm	69.5	1.6	8.6	1.4	18.2	0.7 Al	97.7	2.3
IMJ 27398, reverse, area 3, SA: 300 µm × 300 µm	74.1	–	5.9	1.0	18.4	0.6 Al	100	–
IMJ 27398, reverse, area 4, SA: 80 µm × 80 µm	71.0	–	5.8	0.5	22.1	0.6 Al	100	–
IMJ 27398, obverse, area 1 (Figure 5d, main article), SA: 500 µm × 500 µm	68.5	–	10.6	1.5	18.3	0.8 Al, 0.3 S	100	–
IMJ 27398, obverse, area 2 (Figure 5d, main article), SA: 500 µm × 500 µm	78.1	–	–	0.8	19.9	0.8 Al, 0.4 S	100	–
IMJ 34542, reverse, area 1, SA: 300 µm × 300 µm	73.1	–	5.8	0.8	20.3	–	100	–
IMJ 34542, reverse, area 2, SA: 300 µm × 300 µm	76.4	1.4	–	0.8	20.7	0.7 Al	98.2	1.8

IMJ 34542, reverse, area 3, SA: 300 µm × 300 µm	74.2	1.7	–	0.9	22.4	0.8 Al	97.8	2.2
IMJ 34542, reverse, area 4, SA: 80 µm × 80 µm	76.4	–	–	1.0	21.1	1.5 Al	100	–
IMJ 34542, obverse, area 1, SA: 800 µm × 800 µm	56.6	2.4	21.9	3.0	16.1	–	100	4.1
IMJ 34542, obverse, area 2, SA: 200 µm × 200 µm	69.9	–	8.2	1.0	20.1	0.8 Al	100	–
IMJ 34539, reverse, area 1, SA: 300 µm × 300 µm	81.7	–	3.6	0.5	13.8	0.4 S	100	–
IMJ 34539, reverse, area 2, SA: 300 µm × 300 µm	77.5	–	2.2	–	20.3	–	100	–
IMJ 34539, reverse, area 3, SA: 300 µm × 300 µm	76.3	–	2.8	–	20.9	–	100	–
IMJ 34539, reverse, area 4, SA: 300 µm × 300 µm	91.2	–	–	0.6	7.8	0.4 S	100	–
IMJ 34539, obverse, area 1, SA: 800 µm × 800 µm	70.3	–	10.9	0.6	17.2	0.4 Al, 0.6 Ca	100	–
IMJ 34539, obverse, area 2, SA: 800 µm × 800 µm	70.4	–	10.2	0.6	18.3	0.5 S	100	–
IMJ 34543, reverse, area 1 (Figure S2d), SA: 300 µm × 300 µm	75.6	–	3.4	–	20.3	0.7 Al	100	–
IMJ 34543, reverse, area 2 (Figure S2d), SA: 300 µm × 300 µm	92.4	2.8	–	–	4.8	–	97.1	2.9
IMJ 34543, reverse, area 3 (Figure S2d), SA: 300 µm × 300 µm	86.8	1.9	–	–	11.3	–	97.9	2.1
IMJ 34543, reverse, area 4 (Figure S2d), SA: 300 µm × 300 µm	76.5	–	–	0.4	21.6	1.5 Al	100	–
IMJ 34543, obverse, area 1 (Figure S2e), SA: 500 µm × 500 µm	76.9	–	–	–	22.1	1.0 Al	100	–
IMJ 34543, obverse, area 2 (Figure S2e), SA: 500 µm × 500 µm	76.8	–	–	–	22.0	1.2 Al	100	–
IAA 153976 (coin with exceptional composition), reverse, area 1, SA: 300 µm × 300 µm	41.4	40.3	14.7	1.6	0.6	0.7 S, 0.7 Ca	50.7	49.3
IAA 153976 (coin with exceptional composition), reverse, area 2, SA: 300 µm × 300 µm	46.4	24.6	21.5	4.0	0.5	0.8 S, 2.2 Ca	65.4	34.6
IAA 153976 (coin with exceptional composition), reverse, area 3, SA: 300 µm × 300 µm	91.5	5.5	3.0	–	–	–	94.3	5.7
IAA 153976 (coin with exceptional composition), reverse, area 4, SA: 300 µm × 300 µm	73.7	16.2	8.8	0.3	0.4	0.6 Ca	82.0	18.0
IAA 153976 (coin with exceptional composition), obverse, area 1, SA: 800 µm × 800 µm	23.6	37.4	28.6	5.8	0.9	0.4 P, 0.4 S, 2.9 Ca	38.7	61.3
IAA 153976 (coin with exceptional composition), obverse, area 2, SA: 20 µm × 20 µm	96.7	–	2.5	–	0.8	–	100	–

IAA 153976 (coin with exceptional composition), obverse, area 3, SA: 300 μm × 300 μm	54.7	16.0	24.3	2.8	–	2.2 Ca	77.4	22.6

Ten Type 5 O1/R2 coins were examined in the current study: IAA 101006, IAA 177246, IAA 153977, IMJ 34538, IMJ 34553, IMJ 34537, IMJ 34554, IMJ 34554, IAA 154383, and IMJ 27387. The bright shiny metallic areas of coin IAA 154383 observed by a multi-focal light microscope (LM) (Figure S3a) are the same bright silver metal regions examined using SEM BSE mode (Figure S3b) and detected by EDS as composed of high purity silver alloy (Table S2). The EDS analysis results of ten Yehud *gerah* Type 5 O1/R2 specimens (obverse and reverse surfaces) are presented in Table S2. The composition of coins IAA 154383 and IMJ 27387 was dissimilar to the composition of the other eight coins (Table S2), and thus these two coins were not included in the average value and SD calculations of the Type 5 O1/R2 coins' alloy composition (main article).

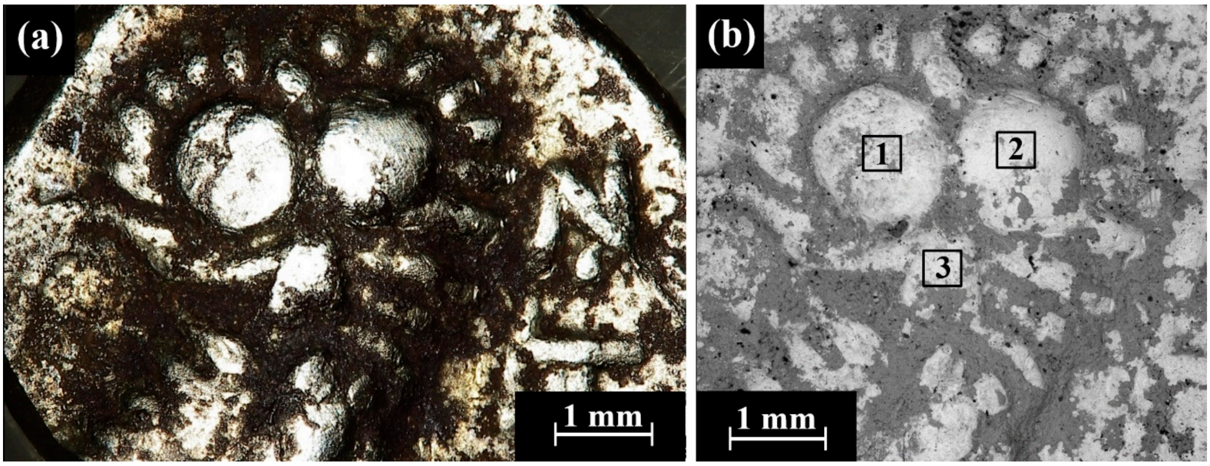

Figure S3. Images of Yehud *gerah* Type 5 O1/R2: (a) coin IAA 154383, reverse (multi-focal LM), showing the face of an owl; and (b) IAA 154383, reverse (SEM, BSE mode), where the bright areas represent shiny silver metal and the dark areas are covered with oxide and corrosion products. The areas inside the squares were examined by EDS analysis.

Table S2. SEM-EDS analysis results of the Yehud *gerah* Type 5 O1/R2 coins, where SA represents the scanned area. Only bright areas according to BSE mode of shiny metal were examined by EDS analysis.

**Sample**	**Composition (wt %)**							
	**Surface**						**Silver alloy**	
	**Ag**	**Cu**	**O**	**Si**	**Cl**	**Others**	**Ag**	**Cu**
IAA 101006, reverse, area 1, SA: 300 μm × 300 μm	85.2	4.7	8.5	0.7	–	0.9 Fe	93.5	6.5
IAA 101006, reverse, area 2, SA: 300 μm × 300 μm	73.9	7.5	16.7	1.9	–	–	90.8	9.2
IAA 101006, reverse, area 3, SA: 300 μm × 300 μm	83.6	2.1	10.8	1.4	0.5	0.5 Ca, 1.1 Fe	97.5	2.5
IAA 101006, reverse, area 4, SA: 300 μm × 300 μm	93.5	0.9	5.0	0.6		–	99.0	1.0
IAA 101006, obverse, area 1, SA: 300 μm × 300 μm	87.2	9.2	–	0.5	1.9	1.2 S	90.5	9.5
IAA 101006, obverse, area 2, SA: 300 μm × 300 μm	81.3	4.6	10.0	1.7	1.0	0.6 Ca, 0.8 Fe	94.6	5.4
IAA 101006, obverse, area 3, SA: 300 μm × 300 μm	77.9	6.7	10.4	0.9	1.6	1.8 S, 0.7 Ca	92.1	7.9

IAA 177246, reverse, area 1 (Figure 6a, main article), SA: 300 µm × 300 µm	81.3	4.4	4.8	0.4	6.5	1.4 S, 1.2 Al	94.9	5.1
IAA 177246, reverse, area 2 (Figure 6a, main article), SA: 300 µm × 300 µm	87.0	4.3	5.0	0.4	1.0	1.3 S, 1.0 Al	95.3	4.7
IAA 177246, reverse, area 3 (Figure 6a, main article), SA: 300 µm × 300 µm	84.4	5.5	6.5	0.8	0.6	1.6 S, 0.6 Al	91.5	8.5
IAA 177246, reverse, area 4 (Figure 6a, main article), SA: 300 µm × 300 µm	89.8	3.8	4.6	–	0.5	1.3 S	95.9	4.1
IAA 177246, obverse, area 1, SA: 500 µm × 500 µm	87.2	4.1	5.3	0.6	1.2	1.6 S	95.5	4.5
IAA 177246, obverse, area 2, SA: 500 µm × 500 µm	83.9	6.0	4.0	–	2.7	1.0 Al, 2.4 S	93.3	6.7
IAA 177246, obverse, area 3, SA: 500 µm × 500 µm	85.1	4.2	5.3	–	2.7	1.1 Al, 1.6 S	95.3	4.7
IAA 153977, reverse, area 1 (Figure 6b, main article), SA: 300 µm × 300 µm	90.4	4.3	5.3	–	–	–	95.5	4.5
IAA 153977, reverse, area 2 (Figure 6b, main article), SA: 300 µm × 300 µm	94.0	2.4	3.6	–	–	–	97.5	2.5
IAA 153977, reverse, area 3 (Figure 6b, main article), SA: 300 µm × 300 µm	77.2	8.4	12.7	0.9	–	0.8 S	90.2	9.8
IAA 153977, obverse, area 1, SA: 300 µm × 300 µm	90.6	3.5	5.2	0.4	–	0.3 Al	96.3	3.7
IAA 153977, obverse, area 2, SA: 300 µm × 300 µm	86.9	3.9	7.7	1.0	0.5	–	95.7	4.3
IAA 153977, obverse, area 3, SA: 300 µm × 300 µm	91.4	3.6	4.4	0.6	–	–	96.2	3.8
IMJ 34538, reverse, area 1, SA: 300 µm × 300 µm	75.5	–	4.8	–	19.1	0.6 Al	100	–
IMJ 34538, reverse, area 2, SA: 300 µm × 300 µm	71.7	3.3	11.2	1.3	11.8	0.7 Al	95.6	4.4
IMJ 34538, reverse, area 3, SA: 300 µm × 300 µm	69.7	3.8	6.7	0.8	18.0	1.0 Al	94.8	5.2
IMJ 34538, reverse, area 4, SA: 300 µm × 300 µm	77.0	2.4	8.7	0.9	10.3	0.7 Al	97.0	3.0
IMJ 34538, obverse, area 1, SA: 300 µm × 300 µm	88.9	1.3	5.0	0.6	4.2	–	98.6	1.4
IMJ 34538, obverse, area 2, SA: 300 µm × 300 µm	77.1	3.7	–	–	19.2	–	95.4	4.6
IMJ 34538, obverse, area 3, SA: 300 µm × 300 µm	77.4	–	3.0	0.5	19.1	–	100	–
IMJ 34553, reverse, area 1 (Figure 6c, main article), SA: 300 µm × 300 µm	92.3	3.9	2.3	1.5	–	–	95.9	4.1

IMJ 34553, reverse, area 2 (Figure 6c, main article), SA: 300 µm × 300 µm	72.5	–	4.2	0.4	20.5	1.1 Au, 1.3 Al	100	–
IMJ 34553, reverse, area 3 (Figure 6c, main article), SA: 300 µm × 300 µm	90.8	2.7	3.0	–	3.5	–	97.1	2.9
IMJ 34553, reverse, area 4 (Figure 6c, main article), SA: 200 µm × 200 µm	89.6	2.2	3.1	0.3	4.8	–	97.6	2.4
IMJ 34553, obverse, area 1, SA: 200 µm × 200 µm	72.5	1.1	3.6	0.4	20.9	1.5 Al	98.5	1.5
IMJ 34553, obverse, area 2, SA: 200 µm × 200 µm	91.6	2.5	2.6	–	3.3	–	97.3	2.7
IMJ 34553, obverse, area 3, SA: 200 µm × 200 µm	69.5	0.8	5.5	1.0	17.8	1.5 Au, 3.9 Al	98.9	1.1
IMJ 34537, reverse, area 1 (Figure 6c, main article), SA: 300 µm × 300 µm	91.3	3.8	2.1	–	2.3	0.5 Al	96.0	4.0
IMJ 34537, reverse, area 2 (Figure 6c, main article), SA: 300 µm × 300 µm	91.4	3.9	2.3	–	2.0	0.4 Al	95.9	4.1
IMJ 34537, reverse, area 3 (Figure 6c, main article), SA: 300 µm × 300 µm	91.8	3.8	2.8	–	1.6	–	96.0	4.0
IMJ 34537, reverse, area 4 (Figure 6c, main article), SA: 300 µm × 300 µm	75.7	–	3.9	0.5	14.8	5.1 Al	100	–
IMJ 34537, obverse, area 1, SA: 300 µm × 300 µm	90.5	2.1	–	–	7.4	–	97.7	2.3
IMJ 34537, obverse, area 2, SA: 300 µm × 300 µm	89.4	2.2	1.9	–	6.5	–	97.6	2.4
IMJ 34537, obverse, area 3, SA: 300 µm × 300 µm	75.4	1.1	6.4	0.4	16.1	0.6 Al	98.6	1.4
IMJ 34554, reverse, area 1, SA: 300 µm × 300 µm	85.5	6.7	7.3	–	0.5	–	92.7	7.3
IMJ 34554, reverse, area 2, SA: 300 µm × 300 µm	92.6	2.9	4.5	–	–	–	97.0	3.0
IMJ 34554, reverse, area 3, SA: 300 µm × 300 µm	92.5	2.2	4.7	–	0.6	–	97.6	2.3
IMJ 34554, reverse, area 4, SA: 300 µm × 300 µm	96.6	–	3.4	–	–	–	100	–
IMJ 34554, obverse, area 1, SA: 300 µm × 300 µm	88.6	1.0	7.6	0.4	2.4	–	98.9	1.1
IMJ 34554, obverse, area 2, SA: 300 µm × 300 µm	88.5	1.9	5.6	0.3	3.7	–	97.9	2.1
IMJ 34554, obverse, area 3, SA: 300 µm × 300 µm	81.8	2.6	12.2	1.2	2.2	–	96.9	3.1
IMJ 34620, reverse, area 1, SA: 300 µm × 300 µm	93.8	4.0	2.2	–	–	–	95.9	4.1

IMJ 34620, reverse, area 2, SA: 300 µm × 300 µm	96.9	3.1	–	–	–	–	96.9	3.1
IMJ 34620, reverse, area 3, SA: 300 µm × 300 µm	94.2	3.5	–	–	0.6	1.7 Pb	96.4	3.6
IMJ 34620, reverse, area 4, SA: 300 µm × 300 µm	75.2	–	2.1	–	21.9	0.8 Al	100	–
IMJ 34620, obverse, area 1, SA: 300 µm × 300 µm	82.2	2.6	3.6	–	10.8	0.8 Al	96.9	3.1
IMJ 34620, obverse, area 2, SA: 300 µm × 300 µm	76.4	–	3.3	–	17.5	2.8 Al	100	–
IMJ 34620, obverse, area 3, SA: 300 µm × 300 µm	77.5	–	3.4	0.4	16.3	2.4 Al	100	–
IAA 154383 (coin with exceptional composition), reverse, area 1 (Figure S3b), SA: 300 µm × 300 µm	83.8	10.1	6.1	–	–	–	89.2	10.8
IAA 154383 (coin with exceptional composition), reverse, area 2 (Figure S3b), SA: 300 µm × 300 µm	93.2	3.0	3.8	–	–	–	96.9	3.1
IAA 154383 (coin with exceptional composition), reverse, area 3 (Figure S3b), SA: 300 µm × 300 µm	85.9	8.7	5.4	–	–	–	90.8	9.2
IAA 154383 (coin with exceptional composition), reverse, area 4 (Figure S3b), SA: 300 µm × 300 µm	85.6	6.0	8.4	–	–	–	93.4	6.6
IAA 154383 (coin with exceptional composition), obverse, area 1, SA: 300 µm × 300 µm	62.3	24.5	12.6	–	0.6	–	71.8	28.2
IAA 154383 (coin with exceptional composition), obverse, area 2, SA: 300 µm × 300 µm	34.5	45.9	16.5	1.7	0.8	0.6 Ca	42.9	57.1
IAA 154383 (coin with exceptional composition), obverse, area 3, SA: 300 µm × 300 µm	50.5	33.9	15.2	0.4	–	–	59.8	40.2
IMJ 27387 (coin with exceptional composition), reverse, area 1, SA: 300 µm × 300 µm	70.7	3.5	5.0	0.8	18.6	1.4 Al	95.3	4.7
IMJ 27387 (coin with exceptional composition), reverse, area 2, SA: 300 µm × 300 µm	71.8	2.4	4.9	0.6	18.7	1.6 Al	96.8	3.2
IMJ 27387 (coin with exceptional composition), reverse, area 3, SA: 300 µm × 300 µm	68.5	1.4	8.4	1.5	17.9	0.6 Fe, 1.7 Al	98.0	2.0
IMJ 27387 (coin with exceptional composition), reverse, area 4, SA: 300 µm × 300 µm	58.4	14.8	8.6	0.7	16.1	1.4 Al	79.8	20.2
IMJ 27387 (coin with exceptional composition), obverse, area 1, SA: 300 µm × 300 µm	34.0	20.0	27.5	6.1	9.2	1.0 Ca, 2.2 Al	63.0	37.0

IMJ 27387 (coin with exceptional composition), obverse, area 2, SA: 300 µm × 300 µm	17.3	41.6	27.5	6.4	5.2	0.4 Ca, 1.6 Al	29.4	70.6
IMJ 27387 (coin with exceptional composition), obverse, area 3, SA: 300 µm × 300 µm	45.8	10.1	24.4	4.4	10.8	0.4 S, 1.2 Ca, 0.7 Fe, 2.2 Al	81.9	18.1

Four Type 5 O1/R3 coins were examined in the current study: IAA 153979, IAA 153978, IMJ 34555, and IMJ 27425. The SEM-EDS elemental mapping of the obverse of coin IAA 153978 (Figure S4) revealed that the bright areas according to the BSE mode (Figure S4a) contained silver metal (Figure S4b-c); the dark grey areas were rich in Cl (Figure S4b, S4f); while the elements Cu and O (Figure S4d–e, respectively) were distributed rather homogeneously.

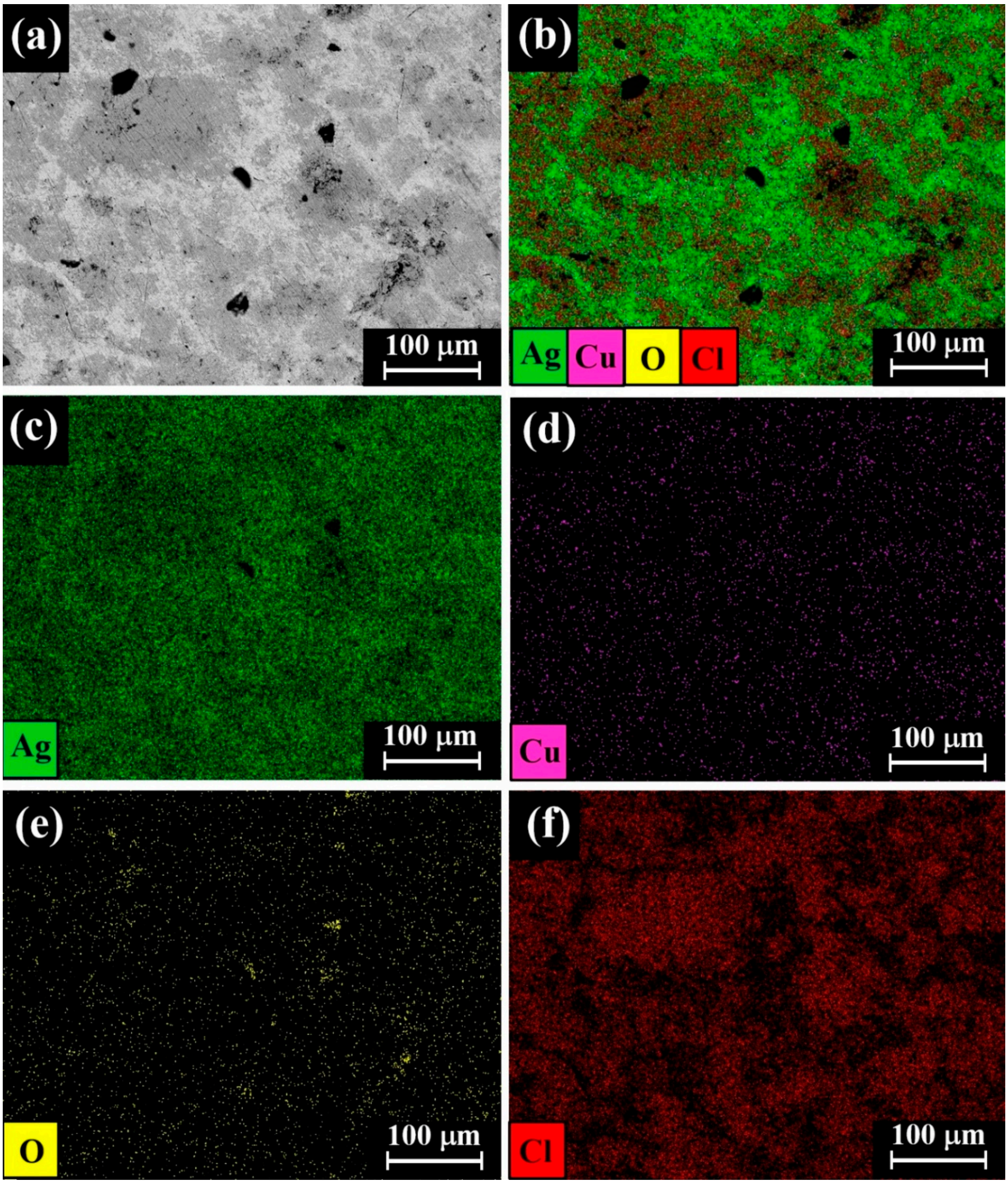

Figure S4. Yehud *gerah* Type 5 O1/R3, IAA 153978 (obverse), SEM–EDS elemental mapping: (a) general view of the examined area; (b) the detected elements (Ag, Ca, O, Cl), where the green areas are rich in silver and red areas are rich in chlorine; (c) presence of Ag; (d) presence of Ca; (e) presence of O; and (f) presence of Cl.

The SEM-EDS analysis results of the surfaces of four Yehud *gerah* Type 5 O1/R3 specimens (obverse and reverse) are presented in Table S3.

Table S3. SEM-EDS analysis results of the Yehud *gerah* Type 5 O1/R3 coins, where SA represents the scanned area. Only bright areas according to BSE mode of shiny metal were examined by EDS analysis.

Sample	Composition (wt %)							
	Surface						Silver alloy	
	Ag	Cu	O	Si	Cl	Others	Ag	Cu
IAA 153979, revers, area 1, SA: 800 μm × 800 μm	67.6	2.2	11.9	–	16.2	2.1 Ca	96.8	3.2
IAA 153979, reverse, area 2, SA: 300 μm × 300 μm	70.1	–	9.0	0.5	17.8	1.8 Al, 0.8 Ca	100	–
IAA 153979, reverse, area 3, SA: 300 μm × 300 μm	.714	7.7	10.3	0.9	7.0	0.5 Al, 2.2 Au	90.3	9.7
IAA 153979, reverse, area 4, SA: 300 μm × 300 μm	70.0	–	8.8	0.6	18.8	1.8 Al	100	–
IAA 153979, reverse, area 5, SA: 300 μm × 300 μm	76.5	–	–	–	19.7	2.3 Al, 1.5 Au	100	–
IAA 153979, obverse, area 1, SA: 300 μm × 300 μm	91.5	7.5	–	–	1.0	–	92.4	7.6
IAA 153979, obverse, area 2, SA: 300 μm × 300 μm	84.2	13.8	–	–	2.0	–	85.9	14.1
IAA 153979, obverse, area 3, SA: 300 μm × 300 μm	81.5	4.5	11.3	–	2.1	0.6 Ca	94.8	5.2
IAA 153978, reverse, area 1 (Figure 7b, main article), SA: 300 μm × 300 μm	69.4	2.6	7.3	1.2	18.9	0.6 Al	96.4	3.6
IAA 153978, reverse, area 2 (Figure 7b, main article), SA: 300 μm × 300 μm	70.6	3.8	5.7	0.8	19.1	–	94.9	5.1
IAA 153978, reverse, area 3 (Figure 7b, main article), SA: 300 μm × 300 μm	68.7	3.9	9.5	1.1	16.3	0.5 Al	94.6	5.4
IAA 153978, reverse, area 4 (Figure 7b, main article), SA: 300 μm × 300 μm	73.2	–	5.2	0.5	20.7	0.4	100	–
IAA 153978, obverse, area 1, SA: 300 μm × 300 μm	72.4	6.3	–	0.8	20.5	–	92.0	8.0
IAA 153978, obverse, area 2, SA: 300 μm × 300 μm	71.1	3.8	6.5	0.6	17.6	0.4 Al	94.9	5.1
IAA 153978, obverse, area 3, SA: 300 μm × 300 μm	78.2	3.8	–	0.9	17.1	–	95.3	4.6
IAA 153978, obverse, area 4, SA: 300 μm × 300 μm	85.2	1.6	–	0.7	12.5	–	98.2	1.8
IMJ 34555, reverse, area 1, SA: 300 μm × 300 μm	72.6	–	5.6	0.7	20.2	0.9 Al	100	–
IMJ 34555, reverse, area 2, SA: 300 μm × 300 μm	70.8	–	10.6	0.9	16.6	1.1 Al	100	–

IMJ 34555, reverse, area 3, SA: 300 μm × 300 μm	69.9	–	9.4	0.8	18.2	1.2 Al, 0.5 Ca	100	–
IMJ 34555, reverse, area 4, SA: 300 μm × 300 μm	69.7	–	9.4	1.0	18.4	1.5 Al	100	–
IMJ 34555, obverse, area 1, SA: 300 μm × 300 μm	63.5	–	16.8	1.8	16.7	1.2 Al	100	–
IMJ 34555, obverse, area 2, SA: 800 μm × 800 μm	66.1	–	12.1	1.6	17.9	1.3 Al, 1.0 Ca	100	–
IMJ 34555, obverse, area 3, SA: 300 μm × 300 μm	69.7	–	9.2	1.2	18.8	1.1 Al	100	–
IMJ 27425, reverse, area 1, SA: 300 μm × 300 μm	82.6	–	7.2	0.5	8.4	1.3 Al	100	–
IMJ 27425, reverse, area 2, SA: 300 μm × 300 μm	88.7	–	7.3	0.9	2.3	0.8 Al	100	–
IMJ 27425, reverse, area 3, SA: 300 μm × 300 μm	84.7	–	10.8	0.7	3.1	0.7 Al	100	–
IMJ 27425, reverse, area 4, SA: 300 μm × 300 μm	95.8	–	–	0.5	2.5	0.6 Al, 0.6 S	100	–
IMJ 27425, obverse, area 1, SA: 300 μm × 300 μm	83.6	–	–	–	15.1	1.3 Al	100	–
IMJ 27425, obverse, area 2, SA: 300 μm × 300 μm	80.0	–	–	0.7	17.8	1.5 Al	100	–
IMJ 27425, obverse, area 3, SA: 300 μm × 300 μm	77.8	–	9.1	0.9	10.5	1.7 Al	100	–
IMJ 27425, obverse, area 4, SA: 300 μm × 300 μm	87.3	–	8.9	0.7	2.6	0.5 Al	100	–

Eight Yehud *gerah* Type 5 O1/R4 coins were examined in the current study: Ramallah area hoard nos. 2–6. IAA 153980, IMJ 34556, and IAA 153981. The SEM-EDS analysis results of eight Type 5 O1/R4 specimens (obverse and reverse surfaces) are presented in Table S4. The composition of coin IAA 153981 was dissimilar to the composition the other seven coins (Table S4), and hence this coin was not included in the average value and SD calculations of the Type 5 O1/R4 alloy composition (main article).

Table S4. SEM-EDS analysis results of the Yehud *gerah* Type 5 O1/R4 coins (each scanned area was 300 μm × 300 μm). Only bright areas according to BSE mode of shiny metal were examined by EDS analysis. RH represents Ramallah Hoard (nos. 2–6).

**Sample**	**Composition (wt %)**							
	**Surface**						**Silver alloy**	
	**Ag**	**Cu**	**O**	**Si**	**Cl**	**Others**	**Ag**	**Cu**
RH2, reverse, area 1 (Figure 8b, main article)	89.5	3.8	3.0	–	3.7	–	95.9	4.1
RH2, reverse, area 2 (Figure 8b, main article)	81.5	1.6	3.4	–	13.0	0.5 Al	98.1	1.9
RH2, reverse, area 3 (Figure 8b, main article)	84.8	2.4	2.8	–	9.3	0.7 Al	97.2	2.8
RH2, reverse, area 4 (Figure 8b, main article)	83.0	1.4	4.1	–	11.5	–	98.3	1.7

RH2, obverse, area 1	72.4	–	5.9	0.5	19.1	2.1 Al	100	–
RH2, obverse, area 2	77.6	–	2.2	0.6	19.6	–	100	–
RH2, obverse, area 3	71.5	0.8	5.2	0.4	19.9	1.6Al, 0.6 Ca	98.9	1.1
RH3, reverse., area 1 (Figure 8c, main article)	73.8	–	4.1	–	20.1	2.0 Al	100	–
RH3, reverse, area 2 (Figure 8c, main article)	76.8	–	2.2	–	21.0	–	100	–
RH3, reverse, area 3 (Figure 8c, main article)	80.9	–	3.6	–	15.5	–	100	–
RH3, obverse, area 1	76.8	–	–	–	20.1	3.1 Al	100	–
RH3, obverse, area 2	76.4	–	–	–	21.3	2.3 Al	100	–
RH3, obverse., area 3	86.9	2.1	–	–	10.6	0.4 Al	97.6	2.4
RH4, reverse, area 1	90.3	2.2	2.9	–	4.6	–	97.6	2.4
RH4, reverse, area 2	82.8	2.1	3.0	–	12.1	–	97.5	2.5
RH4, reverse, area 3	83.0	1.4	3.3	–	12.3	–	98.3	1.7
RH4, reverse, area 4	76.5	0.9	3.6	–	18.2	0.8 Al	98.8	1.2
RH4, obverse, area 1	75.9	0.8	–	–	22.2	1.1 Al	99.0	1.0
RH4, obverse, area 2	77.4	–	–	–	22.9	0.6 Al	100	–
RH4, obverse, area 3	70.6	–	7.0	0.8	19.9	1.7 Al	100	–
RH5, reverse, area 1 (Figure 8d, main article)	66.0	–	17.0	1.6	9.6	0.7 Al, 5.1 Ca	100	–
RH5, reverse, area 2 (Figure 8d, main article)	80.2	–	6.2	1.0	11.5	0.4 Al, 0.7 Ca	100	–
RH5, reverse, area 3 (Figure 8d, main article)	75.2	–	9.5	1.2	12.9	0.8 Al, 0.4 Ca	100	–
RH5, reverse, area 4 (Figure 8d, main article)	71.9	–	13.1	1.5	12.2	0.8 Al, 0.5 Ca	100	–
RH5, obverse, area 1	74.3	–	4.1	0.3	19.8	0.7 Al, 0.8 Ca	100	–
RH5, obverse, area 2	71.7	–	6.1	0.3	19.8	0.6 Al, 1.5 Ca	100	–
RH5, obverse, area 3	75.3	–	3.0	–	20.5	0.6 Al, 0.6 Ca	100	–
RH6, reverse, area 1	77.6	–	4.9	0.9	15.9	0.7 Al	100	–
RH6, reverse, area 2	78.0	–	4.8	0.5	15.2	1.5 Al	100	–
RH6, reverse, area 3	79.5	–	3.4	0.4	15.2	1.5 Al	100	–

RH6, reverse, area 4	72.9	–	5.7	0.9	18.7	1.8 Al	100	–
RH6, obverse, area 1	80.3	–	2.6	–	16.6	0.5 Al	100	–
RH6, obverse, area 2	86.2	–	2.8	–	10.6	0.4 Al	100	–
RH6, obverse, area 3	86.2	2.5	3.4	–	7.4	0.5 Al	97.2	2.8
RH6, obverse, area 4	82.5	1.8	4.5	0.5	10.2	0.5 S	97.9	2.1
IAA 153980, reverse, area 1	84.7	–	5.0	0.4	8.8	0.6 S, 0.5 Al	100	–
IAA 153980, reverse, area 2	77.8	1.3	5.2	0.6	14.3	0.3 S, 0.5 Al	98.4	1.6
IAA 153980, reverse, area 3	76.4	1.3	5.6	0.7	15.2	0.3 S, 0.5 Al	98.3	1.7
IAA 153980, reverse, area 4	81.4	1.6	5.8	0.7	10.1	0.1 S, 0.3 Al	98.1	1.9
IAA 153980, obverse, area 1	82.5	1.8	4.5	0.5	10.2	0.5 S	97.9	2.1
IAA 153980, obverse, area 2	88.3	3.0	3.8	0.6	4.0	0.3 S	96.7	3.3
IAA 153980, obverse, area 3	85.1	2.2	6.2	0.6	5.5	0.4 S	97.5	2.5
IMJ 34556, reverse, area 1	74.6	–	2.6	–	21.1	1.7 Al	100	–
IMJ 34556, reverse, area 2	75.3	–	2.7	–	20.9	1.1 Al	100	–
IMJ 34556, reverse, area 3	73.5	–	3.9	0.5	20.9	1.2 Al	100	–
IMJ 34556, obverse, area 1	74.1	–	4.0	0.3	20.2	1.4 Al	100	–
IMJ 34556, obverse, area 2	72.1	–	6.1	0.5	19.9	1.4 Al	100	–
IMJ 34556, obverse, area 3	72.4	–	5.0	0.4	20.6	1.4 Al, 0.2 S	100	–
IAA 153981 (coin with exceptional composition), reverse, area 1	51.9	11.5	19.3	3.6	13.7	–	81.9	18.1
IAA 153981 (coin with exceptional composition), reverse, area 2	53.6	12.1	19.4	3.6	11.3	–	81.6	18.4
IAA 153981 (coin with exceptional composition), reverse, area 3	62.2	7.2	11.2	2.2	17.2	–	89.6	10.4
IAA 153981 (coin with exceptional composition), reverse, area 4	71.8	1.5	6.7	0.9	19.1	–	98.0	2.0
IAA 153981(coin with exceptional composition), obverse, area 1	86.2	4.7	5.3	0.5	3.3	–	94.8	5.1
IAA 153981 (coin with exceptional composition), obverse, area 2	73.8	7.4	11.2	1.7	5.9	–	90.1	9.1
IAA 153981 (coin with exceptional composition), obverse, area 3	59.3	10.2	13.5	2.6	14.4	–	85.3	14.7

Two Yehud *gerah* Type 5 O1/R5 coins were examined in the current study: IMJ 34558 and IMJ 27388. The SEM-EDS analysis results of the Type 5 O1/R5 specimens (obverse and reverse surfaces) are presented in Table S5.

Table S5. SEM-EDS analysis results of the Yehud *gerah* Type 5 O1/R5 coins. Only bright areas according to BSE mode of shiny metal were examined by EDS analysis.

**Sample**	**Composition (wt %)**							
	**Surface**						**Silver alloy**	
	**Ag**	**Cu**	**O**	**Si**	**Cl**	**Others**	**Ag**	**Cu**
IMJ 34558, reverse, area 1 (Figure 9a, main article), SA: 300 μm × 300 μm	69.1	–	10.4	1.2	18.2	1.1 Al	100	–
IMJ 34558, reverse, area 2 (Figure 9a, main article), SA: 300 μm × 300 μm	68.5	–	11.6	1.5	15.7	1.3 Al, 0.8 S, 0.6 Ca	100	–
IMJ 34558, reverse, area 3 (Figure 9a, main article), SA: 300 μm × 300 μm	61.9	1.3	16.4	1.8	15.8	1.8 Al, 0.4 S, 0.6 Ca	97.9	2.1
IMJ 34558, reverse, area 4 (Figure 9a, main article), SA: 300 μm × 300 μm	71.0	–	9.5	0.8	17.3	1.4 Al	100	–
IMJ 34558, obverse, area 1, SA: 300 μm × 300 μm	69.7	–	9.4	1.5	17.4	1.5 Al, 0.5 S	100	–
IMJ 34558, obverse, area 2, SA: 300 μm × 300 μm	66.2	1.6	13.7	2.1	15.6	0.8 S	97.6	2.4
IMJ 34558, obverse, area 3, SA: 200 μm × 200 μm	77.0	–	–	0.6	21.8	0.6 Al	100	–
IMJ 27388, reverse, area 1, SA: 300 μm × 300 μm	93.9	–	4.5	–	1.6	–	100	–
IMJ 27388, reverse, area 2, SA: 300 μm × 300 μm	88.1	–	6.0	0.8	2.0	3.1 S	100	–
IMJ 27388, reverse, area 3, SA: 300 μm × 300 μm	90.2	–	5.6	0.3	1.9	2.0 S	100	–
IMJ 27388, reverse, area 4, SA: 300 μm × 300 μm	95.4	–	–	0.4	3.7	0.5 Al	100	–
IMJ 27388, obverse, area 1, SA: 800 μm × 800 μm	87.3	–	8.9	0.7	2.6	0.5 Al	100	–
IMJ 27388, obverse, area 2, SA: 300 μm × 300 μm	87.4	–	9.0	0.7	2.9	–	100	–

IMJ 27388, obverse, area 3, SA: 300 µm × 300 µm	97.7	–	–	0.5	1.8	–			100	–

2. Yehud Half *Gerah* Type 16 O2/R2

Nine Yehud half *gerah* Type 16 O2/R2 coins were examined in the current study: Edom hoard nos. 1–6, IMJ 27383, IMJ 27414, and IMJ 34566. The SEM images of the Type 16 O2/R2 coins (obverse with Persian king wearing a jagged crown and reverse with a falcon in flight) shows the areas (inside the squares) that were examined by EDS analysis (Figure S5).

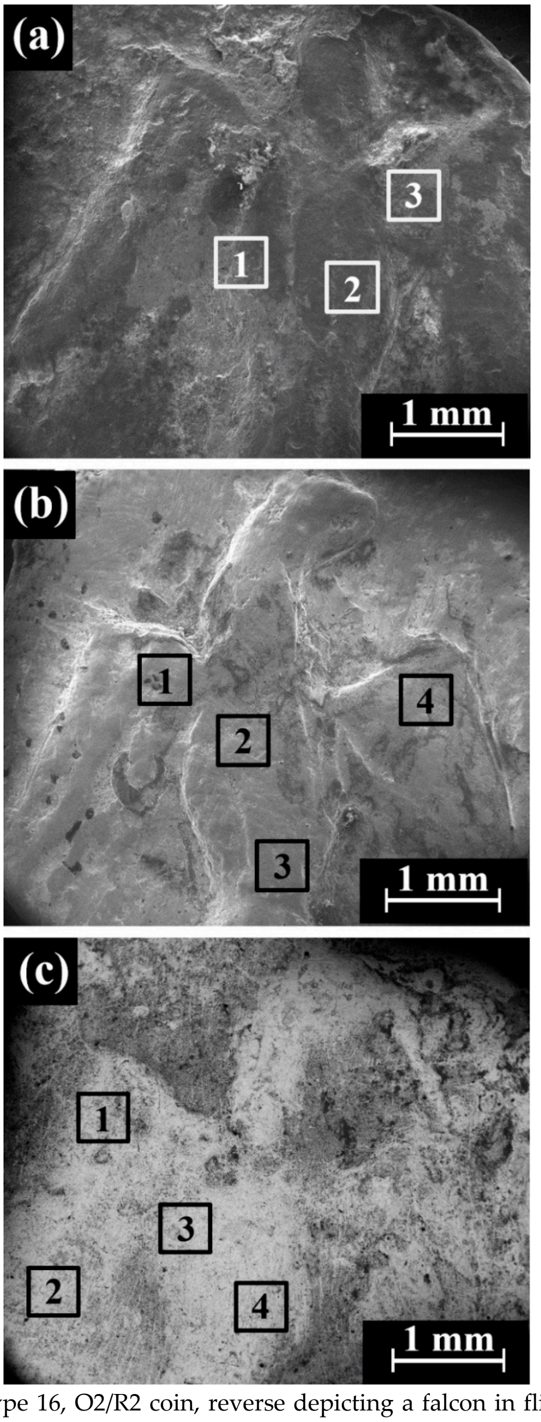

Figure S5. Yehud half *gerah* Type 16, O2/R2 coin, reverse depicting a falcon in flight: (a) Edom hoard no. 4 (SE mode); (b) Edom hoard no. 5 (SE mode); and (c) Edom hoard no. 6 (BSE mode), where the brighter areas according to the BSE mode are better preserved than the darker areas. The areas inside the squares were examined by EDS analysis.

The SEM-EDS analysis results of nine Type 16 O2/R2 specimens (obverse and reverse surfaces) are presented in Table S6.

Table S6. SEM-EDS analysis results of the Yehud half *gerah* Type 16 O2/R2 coins. Only bright areas according to BSE mode of shiny metal were examined by EDS analysis.

Sample	Composition (wt %)							
	Surface						Silver alloy	
	Ag	Cu	O	Si	Cl	Others	Ag	Cu
Edom hoard no. 1, reverse, area 1, SA: 500 μm × 500 μm	78.1	–	8.2	0.6	10.1	1.3 Al, 0.9 Fe, 0.8 Au	100	–
Edom hoard no. 1, reverse, area 2, SA: 300 μm × 300 μm	86.3	–	–	–	12.4	1.3 Al	100	–
Edom hoard no. 1, reverse, area 3, SA: 300 μm × 300 μm	93.4	–	–	–	5.9	0.7 Al	100	–
Edom hoard no. 1, reverse, area 4, SA: 300 μm × 300 μm	85.3	–	4.0	–	9.6	1.1 Al	100	–
Edom hoard no. 1, reverse, area 5, SA: 300 μm × 300 μm	85.5	–	5.7	–	7.6	1.2 Al	100	–
Edom hoard no. 1, obverse, area 1, SA: 500 μm × 300 μm	84.3	–	4.2	–	9.8	1.7 Al	100	–
Edom hoard no. 1, obverse, area 2, SA: 500 μm × 300 μm	86.6	–	–	–	11.6	1.8 Al	100	–
Edom hoard no. 2, reverse (Figure 10b, main article), SA: 500 μm × 500 μm	94.3	–	–	–	4.3	1.4 S	100	–
Edom hoard no. 2, reverse, area 2 (Figure 10b, main article), SA: 300 μm × 300 μm	93.4	1.1	2.7	–	2.2	0.6 Al	98.8	1.2
Edom hoard no. 2, reverse, area 3 (Figure 10b, main article), SA: 300 μm × 300 μm	97.5	–	–	–	1.9	0.6 Al	100	–
Edom hoard no. 2, reverse, area 4 (Figure 10b, main article), SA: 300 μm × 300 μm	91.4	1.2	–	–	3.6	1.4 Al, 0.4 S, 2.0 Pb	98.7	1.3
Edom hoard no. 2, reverse, area 5 (Figure 10b, main article), SA: 300 μm × 300 μm	94.0	–	–	–	4.2	1.8 Al	100	–
Edom hoard no. 2, obverse, area 1, SA: 500 μm × 500 μm	95.6	–	–	–	3.0	1.4 Al	100	–
Edom hoard no. 2, obverse, area 2, SA: 500 μm × 500 μm	94.1	1.1	–	–	2.8	1.3 Al, 0.7 S	98.8	1.2
Edom hoard no. 3, reverse, area 1 (Figure 10c, main article), SA: 200 μm × 200 μm	97.0	–	3.0	–	–	–	100	–
Edom hoard no. 3, reverse, area 2 (Figure 10c, main article), SA: 300 μm × 300 μm	95.1	–	3.4	–	1.0	0.5 Al	100	–

Edom hoard no. 3, reverse, area 3 (Figure 10c, main article), SA: 300 µm × 300 µm	87.9	–	5.0	–	4.2	2.4 Al, 0.5 S	100	–
Edom hoard no. 3, reverse, area 4 (Figure 10c, main article), SA: 300 µm × 300 µm	83.8	–	7.9	–	3.8	2.1 Al, 1.1 S, 1.3 Ca	100	–
Edom hoard no. 3, obverse, area 1, SA: 500 µm × 500 µm	82.1	–	8.1	–	5.6	1.6 Al, 0.6 S, 2.0 Ca	100	–
Edom hoard no. 3, obverse, area 2, SA: 500 µm × 500 µm	85.2	–	7.4	–	5.6	1.8 Al	100	–
Edom hoard no. 4, reverse, area 1 (Figure S5a), SA: 300 µm × 300 µm	62.4	–	15.7	0.9	13.3	5.6 Al, 2.1 Ca	100	–
Edom hoard no. 4, reverse, area 2 (Figure S5a), SA: 200 µm × 200 µm	65.0	–	11.1	0.8	14.1	6.3 Al, 2.7 Ca	100	–
Edom hoard no. 4, reverse, area 3 (Figure S5a), SA: 250 µm × 250 µm	62.8	1.1	11.9	1.2	13.1	6.2 Al, 3.7 Ca	98.3	1.7
Edom hoard no. 4, obverse, area 1, SA: 200 µm × 200 µm	67.3	–	8.0	–	14.7	6.8 Al, 3.2 Ca	100	–
Edom hoard no. 4, obverse, area 2, SA: 200 µm × 200 µm	43.0	–	25.4	1.2	9.6	4.8 Al, 16 Ca	100	–
Edom hoard no. 5, reverse, area 1 (Figure S5b), SA: 300 µm × 300 µm	80.0	–	12.7	1.1	4.0	1.2 Al, 1.0 S	100	–
Edom hoard no. 5, reverse, area 2 (Figure S5b), SA: 300 µm × 300 µm	95.1	–	–	0.7	2.7	0.9 Al, 0.6 S	100	–
Edom hoard no. 5, reverse, area 3 (Figure S5b), SA: 300 µm × 300 µm	81.3	–	11.0	1.3	2.8	0.7 Al, 2.9 S	100	–
Edom hoard no. 5, reverse, area 4 (Figure S5b), SA: 300 µm × 300 µm	87.9	–	7.9	0.7	1.6	0.6 Al, 1.3 S	100	–
Edom hoard no. 5, obverse, area 1, SA: 300 µm × 300 µm	96.3	–	–	–	3.0	0.7 Al	100	–
Edom hoard no. 5, obverse, area 2, SA: 300 µm × 300 µm	97.8	–	–	–	1.8	0.4 Al	100	–
Edom hoard no. 5, obverse, area 3, SA: 300 µm × 300 µm	74.8	–	13.6	1.9	4.9	1.4 Al, 1.7 S, 1.7 Ca	100	–
Edom hoard no. 6, reverse, area 1 (Figure S5c), SA: 300 µm × 300 µm	69.7	–	12.9	1.3	13.3	0.7 Al, 2.1 Ca	100	–
Edom hoard no. 6, reverse, area 2 (Figure S5c), SA: 300 µm × 300 µm	77.2	–	7.6	0.9	13.2	0.5 Al, 0.6 Ca	100	–
Edom hoard no. 6, reverse, area 3 (Figure S5c), SA: 300 µm × 300 µm	75.6	–	11.0	1.0	11.3	0.5 Al, 0.6 Ca	100	–
Edom hoard no. 6, reverse, area 4 (Figure S5c), SA: 300 µm × 300 µm	72.8	–	14.3	1.6	10.6	0.7 Al	100	–
Edom hoard no. 6, obverse, area 1, SA: 300 µm × 300 µm	74.6	–	10.1	0.8	13.0	1.5 Ca	100	–
Edom hoard no. 6, obverse, area 2, SA: 300 µm × 300 µm	72.6	–	12.9	1.3	10.4	0.6 Al, 0.6 K, 1.0 Ca, 0.6 Fe	100	–
Edom hoard no. 6, obverse, area 3, SA: 300 µm × 300 µm	72.1	–	14.4	1.4	10.3	0.7 Al, 1.1 Ca	100	–
IMJ 27383, reverse, area 1, SA: 300 µm × 300 IMJ µm	92.2	–	6.6	0.6	–	0.6 S	100	–

IMJ 27383, reverse, area 2, SA: 300 µm × 300 µm	93.9	–	5.7	0.4	–	–	100	–
IMJ 27383, reverse, area 3, SA: 300 µm × 300 µm	91.9	–	6.1	0.7	0.6	0.7 S	100	–
IMJ 27383, reverse, area 4, SA: 300 µm × 300 µm	92.4	–	6.0	0.6	0.4	0.6 S	100	–
IMJ 27383, obverse, area 1, SA: 300 µm × 300 µm	82.5	1.2	12.7	0.7	1.4	0.4 Al, 0.4 P, 0.7 S	98.6	1.4
IMJ 27383, obverse, area 2, SA: 300 µm × 300 µm	84.3	–	12.1	1.0	1.2	0.7 Al, 0.7 S	100	–
IMJ 27383, obverse, area 3, SA: 300 µm × 300 µm	98.8	–	–	0.7	0.5	–	100	–
IMJ 27414, reverse, area 1, SA: 300 µm × 300 µm	83.2	–	14.2	1.9	–	0.7 Al	100	–
IMJ 27414, reverse, area 2, SA: 300 µm × 300 µm	89.5	–	9.0	0.6	–	0.5 Al, 0.4 S	100	–
IMJ 27414, reverse, area 3, SA: 300 µm × 300 µm	98.5	–	–	1.0	–	0.5 Al	100	–
IMJ 27414, reverse, area 4, SA: 300 µm × 300 µm	88.0	–	10.4	1.1	–	0.5 Al	100	–
IMJ 34566, reverse, area 1, SA: 300 µm × 300 µm	62.0	–	22.8	3.9	–	0.5 Mg, 1.9 Al, 5.5 S, 2.4 Ca, 1.0 Fe	100	–
IMJ 34566, reverse, area 2, SA: 200 µm × 200 µm	83.1	–	13.3	1.2	0.6	0.5 Al, 1.3 Ca	100	–
IMJ 34566, reverse, area 3, SA: 300 µm × 300 µm	83.8	–	4.2	–	9.8	1.7 Al, 0.5 S	100	–

3. Yehud Quarter *Obol* Type 24 O1/R2

Three Yehud Attic standard quarter *obol* Type 24 O1/R2 coins were examined in the current study: Trans-Jordan hoard nos. 11, 12, 13. The SEM images of these coins (observe with a portrait and reverse with a falcon) shows the areas (inside the squares) that were examined by EDS analysis (Figure S6).

The SEM-EDS analysis results of Type 24 O1/R2 coins (obverse and reverse surfaces) are presented in Table S7.

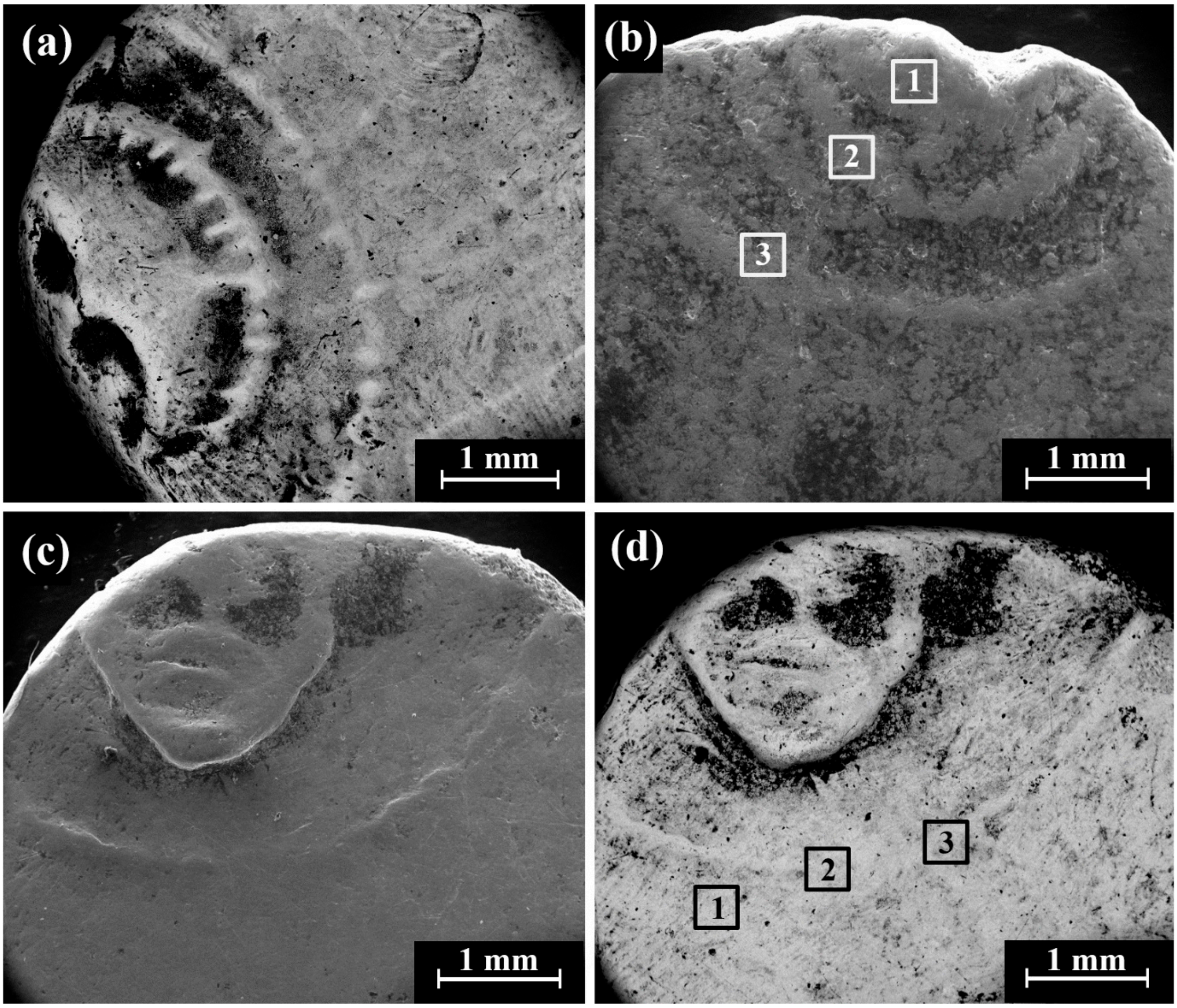

Figure S6. SEM images of the Trans-Jordan hoard Type 24 O1/R2, obverse depicting a facing head: (a) coin Trans-Jordan hoard no. 11 (SE mode); (b) coin Trans-Jordan hoard no. 13 (SE mode); (c,d) coin Trans-Jordan hoard no. 12 (SE mode and BSE mode, respectively). The areas inside the squares were examined by EDS analysis.

Table S7. SEM-EDS analysis results of the Yehud quarter *obol* Type 24 O1/R2 coins, where SA represents the scanned area. Only bright areas according to BSE mode of shiny metal were examined by EDS analysis.

**Sample**	**Composition (wt %)**							
	**Surface**						**Silver alloy**	
	**Ag**	**Cu**	**O**	**Si**	**Cl**	**Others**	**Ag**	**Cu**
Trans-Jordan hoard no. 11, obverse, area 1, SA: 300 μm × 300 μm	75.1	–	8.1	0.7	13.5	2.6 Al	100	–
Trans-Jordan hoard no. 11, obverse, area 2, SA: 300 μm × 300 μm	81.4	–	–	0.8	14.7	3.1 Al	100	–
Trans-Jordan hoard no. 11, obverse, area 3, SA: 300 μm × 300 μm	82.7	–	–	0.6	13.7	3.0 Al	100	–
Trans-Jordan hoard no. 11, reverse, area 1, SA: 300 μm × 300 μm	99.6	–	–	0.4	–	–	100	–
Trans-Jordan hoard no. 11, reverse, area 2, SA: 300 μm × 300 μm	94.6	–	5.0	0.4	–	–	100	–

Trans-Jordan hoard no. 11, reverse, area 3, SA: 300 μm × 300 μm	93.0	–	6.6	0.4	–	–	100	–
Trans-Jordan hoard no. 11, reverse, area 4, SA: 300 μm × 300 μm	92.3	–	7.0	0.7	–	–	100	–
Trans-Jordan hoard no. 12, obverse, area 1, SA: 300 μm × 300 μm	86.5	–	4.0	–	6.9	2.6 Al	100	–
Trans-Jordan hoard no. 12, obverse, area 2, SA: 300 μm × 300 μm	92.9	–	–	0.7	4.4	2.0 Al	100	–
Trans-Jordan hoard no. 12, obverse, area 3, SA: 300 μm × 300 μm	96.5	–	–	0.4	2.0	1.1 Al	100	–
Trans-Jordan hoard no. 12, reverse, area 1, SA: 300 μm × 300 μm	89.0	–	6.6	–	2.4	1.1 Al, 0.9 Ca	100	–
Trans-Jordan hoard no. 12, reverse, area 2, SA: 300 μm × 300 μm	90.5	–	–	–	5.8	3.7	100	–
Trans-Jordan hoard no. 12, reverse, area 3, SA: 300 μm × 300 μm	71.5	–	9.8	0.5	12.2	2.6 Al, 3.4 Ca	100	–
Trans-Jordan hoard no. 12, reverse, area 4, SA: 300 μm × 300 μm	67.7	–	12.9	0.6	14.7	3.2 Al, 0.9 Ca	100	–
Trans-Jordan hoard no. 13, obverse, area 1, SA: 300 μm × 300 μm	72.4	–	8.5	–	17.3	1.8 Ca	100	–
Trans-Jordan hoard no. 13, obverse, area 2, SA: 300 μm × 300 μm	71.6	–	9.3	1.4	17.7	–	100	–
Trans-Jordan hoard no. 13, obverse, area 3, SA: 300 μm × 300 μm	76.7	–	–	1.3	18.4	2.9 Al, 0.7 Fe	100	–
Trans-Jordan hoard no. 13, reverse, area 1, SA: 300 μm × 300 μm	70.9	–	8.6	–	16.4	1.1 Al, 3.0 Ca	100	–
Trans-Jordan hoard no. 13, reverse, area 2, SA: 300 μm × 300 μm	73.5	–	6.1	0.4	17.9	1.4 Al, 0.7 Ca	100	–
Trans-Jordan hoard no. 13, reverse, area 3, SA: 300 μm × 300 μm	79.7	–	–	0.5	18.0	1.8 Al	100	–

4. Yehud *Hemibol* Type 31 O1/R1

Six Yehud *hemibol* Type 31 O1/R1 coins were examined in the current study: IMJ 34631, IMJ 34593, IMJ 34591, IMJ 34709, IMJ 34594, and IMJ 34715. The SEM images of these coins (observe with a portrait and reverse with a falcon) shows the areas (inside the squares) that were examined by EDS analysis (Figure S7).

The SEM-EDS analysis results of Type 31 O1/R1 specimens (obverse and reverse surfaces) are presented in Table S8.

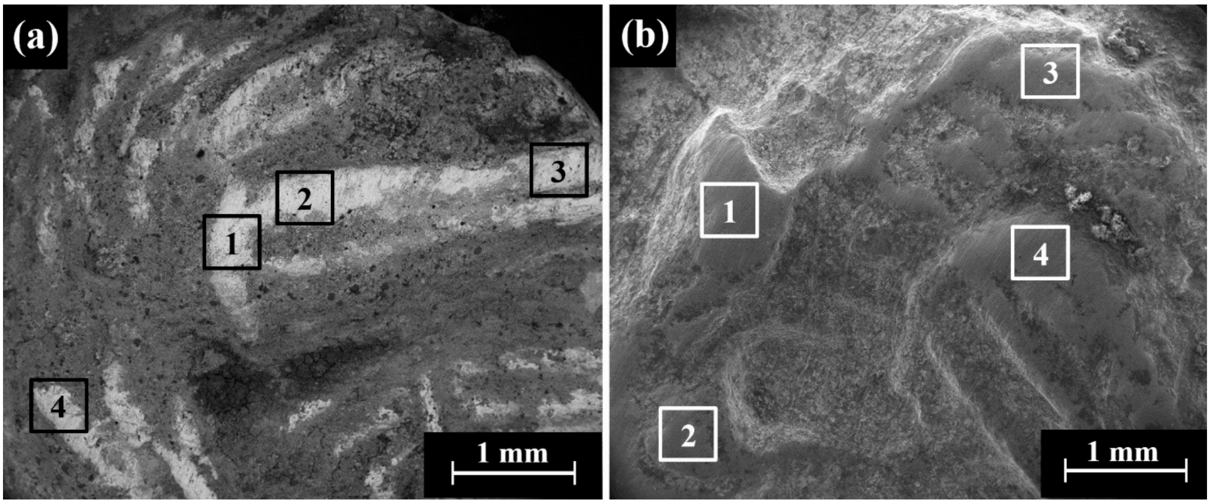

Figure S7. SEM images of the Yehud *hemiobol* Type 31, O1/R1, reverse depicting a bird standing right, head reverted: (a) IMJ 34593 (BSE mode); and (b) IMJ 34591 (SE mode). The areas inside the squares were examined by EDS analysis.

Table S8. SEM-EDS analysis results of the Yehud *hemiobol* Type 31 O1/R1 coins, where SA represents the scanned area. Only bright areas according to BSE mode of shiny metal were examined by EDS analysis.

**Sample**	**Composition (wt %)**							
	**Surface**						**Silver alloy**	
	**Ag**	**Cu**	**O**	**Si**	**Cl**	**Others**	**Ag**	**Cu**
IMJ 34631, reverse, area 1 (Figure 12c, main article), SA: 500 μm × 500 μm	87.1	2.0	4.9	0.4	5.6	–	97.7	2.3
IMJ 34631, reverse, area 2 (Figure 12c, main article), SA: 500 μm × 500 μm	87.2	1.9	4.9	–	6.0	–	97.9	2.1
IMJ 34631, reverse, area 3 (Figure 12c, main article), SA: 300 μm × 300 μm	88.7	1.1	5.1	0.4	4.7	–	98.8	1.2
IMJ 34631, reverse, area 4 (Figure 12c, main article), SA: 300 μm × 300 μm	79.6	1.1	12.1	0.7	5.3	1.2 Al	98.6	1.4
IMJ 34631, obverse, area 1, SA: 500 μm × 500 μm	80.1	1.2	5.4	–	11.7	1.6 Al	98.5	1.5
IMJ 34631, obverse, area 2, SA: 1000 μm × 1000 μm	77.7	1.0	9.1	0.8	9.9	1.5 Al	98.7	1.3
IMJ 34631, obverse, area 3, SA: 300 μm × 300 μm	82.7	0.8	4.2	–	11.7	0.6 Al	99.0	1.0
IMJ 34593, reverse, area 1 (Figure S7a), SA: 500 μm × 500 μm	75.9	7.5	15.0	0.7	–	0.3 Al, 0.6 S	91.0	9.0
IMJ 34593, reverse, area 2 (Figure S7a), SA: 200 μm × 200 μm	85.4	7.3	7.0	–	–	0.3 S	92.1	7.9
IMJ 34593, reverse, area 3 (Figure S7a), SA: 400 μm × 400 μm	74.7	7.1	16.9	0.9	–	0.4 S	91.3	8.7
IMJ 34593, reverse, area 4 (Figure S7a), SA: 400 μm × 400 μm	64.5	14.8	19.1	0.8	–	0.8 S	81.3	18.7

IMJ 34593, obverse, area 1, SA: 500 µm × 500 µm	84.0	8.1	7.1	0.2	0.3	0.3 S	91.2	8.8
IMJ 34593, obverse area 2, SA: 500 µm × 500 µm	80.6	5.8	12.4	0.5	0.3	0.4 S	93.3	6.7
IMJ 34593, obverse, area 3, SA: 500 µm × 500 µm	82.9	4.7	11.9	0.3	0.2	–	94.6	5.4
IMJ 34591, reverse, area 1 (Figure S7b), SA: 300 µm × 300 µm	72.8	–	7.5	0.6	18.4	0.7 Al	100	–
IMJ 34591, reverse, area 2 (Figure S7b), SA: 300 µm × 300 µm	69.4	–	11.3	1.2	17.1	1.0 Al	100	–
IMJ 34591, reverse, area 3 (Figure S7b), SA: 400 µm × 400 µm	71.2	–	9.1	0.5	18.1	0.5 Al, 0.6 Ca	100	–
IMJ 34591, reverse, area 4 (Figure S7b), SA: 200 µm × 200 µm	74.5	–	6.1	0.6	18.0	0.8 Al	100	–
IMJ 34591, obverse, area 1, SA: 500 µm × 500 µm	68.1	–	11.3	1.3	18.2	1.1 Al	100	–
IMJ 34591, obverse, area 2, SA: 300 µm × 300 µm	66.8	–	12.4	2.0	17.0	1.2 Al, 0.6 Fe	100	–
IMJ 34591, obverse, area 3, SA: 100 µm × 100 µm	72.6	–	7.5	1.2	17.6	1.1 Al	100	–
IMJ 34591, reverse, area 4, SA: 100 µm × 100 µm	71.1	–	10.7	1.2	15.4	1.0 Al, 0.6 Fe	100	–
IMJ 34709, reverse, area 1 (Figure 12a, main article), SA: 400 µm × 400 µm	86.4	–	5.1	0.4	7.7	0.4 Al	100	–
IMJ 34709, reverse, area 2 (Figure 12a, main article), SA: 300 µm × 300 µm	81.2	–	6.6	0.6	11.2	0.4 Al	100	–
IMJ 34709, reverse, area 3 (Figure 12a, main article), SA: 300 µm × 300 µm	79.7	–	8.8	0.5	9.2	0.9 Al, 0.9 Ca	100	–
IMJ 34709, reverse, area 4 (Figure 12a, main article), SA: 200 µm × 200 µm	90.3	–	8.0	0.6	0.7	0.4	100	–
IMJ 34709, obverse, area 1, SA: 500 µm × 500 µm	62.9	–	15.9	1.7	15.0	1.5 Al, 0.4 S, 1.9 Ca, 0.7 Fe	100	–
IMJ 34709, obverse, area 2, SA: 300 µm × 300 µm	75.0	–	8.0	0.6	14.8	0.9 Al, 0.7 Ca	100	–
IMJ 34709, obverse, area 3, SA: 200 µm × 200 µm	77.8	–	5.7	0.5	15.4	0.6 Al	100	–
IMJ 34594, reverse, area 1 (Figure 12d, main article), SA: 1000 µm × 1000 µm	78.8	–	8.8	–	11.2	1.2 Al	100	–
IMJ 34594, reverse, area 2 (Figure 12d, main article), SA: 400 µm × 400 µm	79.3	–	8.4	–	8.9	2.8 Al, 0.6 Ca	100	–
IMJ 34594, reverse, area 3 (Figure 12d, main article), SA: 400 µm × 400 µm	71.2	–	19.1	1.6	5.5	0.6 Al, 2.0 Ca	100	–

IMJ 34594, reverse, area 4 (Figure 12d, main article), SA: 400 μm × 400 μm	84.2	–	12.1	0.6	0.8	0.4 Al, 1.9 Au	100	–
IMJ 34594, obverse, area 1, SA: 500 μm × 500 μm	88.4	–	6.5	–	4.3	0.8 Al	100	–
IMJ 34594, obverse, area 2, SA: 1000 μm × 1000 μm	69.5	–	16.9	1.5	9.9	2.2 Al	100	–
IMJ 34594, obverse, area 3, SA: 500 μm × 500 μm	85.5	–	8.9	0.5	4.3	0.8 Al	100	–
IMJ 34715, reverse, area 1, SA: 300 μm × 300 μm	94.5	–	4.8	–	0.7	–	100	–
IMJ 34715, reverse, area 2, SA: 200 μm × 200 μm	91.7	–	7.3	–	0.6	0.4 Ca	100	–
IMJ 34715, reverse, area 3, SA: 500 μm × 500 μm	65.8	–	19.5	1.0	12.3	0.8 Al, 0.6 Ca	100	–
IMJ 34715, reverse, area 4, SA: 400 μm × 400 μm	82.0	–	11.9	0.6	5.5	–	100	–
IMJ 34715, obverse, area 1 (Figure 12b, main article), SA: 500 μm × 500 μm	91.7	–	6.9	0.5	0.9	–	100	–
IMJ 34715, obverse, area 2 (Figure 12b, main article), SA: 1000 μm × 1000 μm	93.8	–	5.5	0.4	0.3	–	100	–
IMJ 34715, obverse, area 3 (Figure 12b, main article), SA: 200 μm × 200 μm	85.8	–	10.1	0.7	2.5	0.5 Al, 0.4 Ca	100	–
IMJ 34715, obverse, area 4 (Figure 12b, main article), SA: 200 μm × 200 μm	90.8	–	6.7	0.7	1.8	–	100	–

5. Bulk Analysis of the Locally Ground Yehud Coins

In order to determine whether the bright metallic areas at the surface of the coins represent the bulk metal, seven representative Yehud silver coins were locally ground with 240–320 silicon carbide grit papers to expose their bulk metal: IAA 153976 and IMJ 27424 (Type 5 O1/R1), IAA 101006 and IAA 154383 (Type 5 O1/R2), IMJ 27383 (Type 16 O2/R2), Trans-Jordan hoard no. 11 (Type 24 O1/R2), and IMJ 34591 (Type 31 O1/R1) (Table S9). Next the coins were cleaned with ethanol and dried and then were examined by SEM-EDS analysis.

Table S9. SEM-EDS analysis results of the Yehud IAA 153976, IMJ 27424, IAA 101006, IAA 154383, IMJ 27383, Trans-Jordan hoard no. 11, and IMJ 34591 coins after roughly grinding the surface.

**Sample**	**Composition (wt %)**							
	**Surface**						**Silver alloy**	
	**Ag**	**Cu**	**O**	**Si**	**Cl**	**Others**	**Ag**	**Cu**
IAA 153976 (coin with exceptional composition), reverse, area 1, SA: 150 μm × 150 μm	52.4	34.1	13.1	–	0.4	–	60.5	39.5
IAA 153976 (coin with exceptional composition), reverse, area 2, SA: 150 μm × 150 μm	55.8	23.0	17.1	–	2.5	1.6 Ca	70.8	29.2

IAA 153976 (coin with exceptional composition), reverse, area 3, SA: 150 µm × 150 µm	59.8	24.2	14.5	–	0.7	0.5 Ca, 0.3 S	71.2	28.8
IAA 153976 (coin with exceptional composition), obverse, area 1, SA: 150 µm × 150 µm	43.1	20.3	32.5	–	0.6	3.5 Ca	68.0	32.0
IAA 153976 (coin with exceptional composition), obverse, area 2, SA: 150 µm × 150 µm	34.0	18.5	39.1	–	0.4	7.5 Ca, 0.5 P	64.8	35.2
IAA 153976 (coin with exceptional composition), obverse, area 3, SA: 150 µm × 150 µm	46.3	21.0	30.8	–	–	1.9 Ca	68.8	31.2
IMJ 27424, reverse, area 1, SA: 150 µm × 150 µm	91.6	3.0	5.4	–	–	–	96.8	3.2
IMJ 27424, reverse, area 2, SA: 150 µm × 150 µm	94.7	1.0	4.3	–	–	–	99.0	1.0
IMJ 27424, reverse, area 3, SA: 150 µm × 150 µm	96.7	–	3.3	–	–	–	100	–
IMJ 27424, obverse, area 1, SA: 150 µm × 150 µm	93.6	0.9	5.5	–	–	–	99.0	1.0
IMJ 27424, obverse, area 3, SA: 150 µm × 150 µm	92.4	1.1	6.0	–	0.5	–	98.8	1.2
IMJ 27424, obverse, area 4, SA: 150 µm × 150 µm	91.9	1.0	6.5	–	0.6	–	98.9	1.1
IAA 101006, reverse, area 1, SA: 150 µm × 150 µm	84.6	4.7	10.7	–	–	–	94.7	5.3
IAA 101006, reverse, area 2, SA: 150 µm × 150 µm	83.7	5.0	10.9	–	0.4	–	94.4	5.6
IAA 101006, reverse, area 3, SA: 150 µm × 150 µm	66.0	8.3	21.4	–	0.7	1.2 Ca, 2.4 Pb	88.8	11.2
IAA 101006, obverse, area 1, SA: 150 µm × 150 µm	75.4	10.6	12.8	–	0.4	0.8 Ca	87.8	12.3
IAA 101006, obverse, area 2, SA: 150 µm × 150 µm	76.9	9.2	11.6	–	0.5	1.8 Pb	89.3	10.7
IAA 101006, obverse, area 3, SA: 150 µm × 150 µm	83.9	10.6	5.5	–	–	–	88.8	11.2
IAA 154383 (coin with exceptional composition), reverse, area 1, SA: 150 µm × 150 µm	86.5	8.5	5.0	–	–	–	91.1	8.9
IAA 154383 (coin with exceptional composition), reverse, area 2, SA: 150 µm × 150 µm	85.2	9.0	5.8	–	–	–	90.4	9.6
IAA 154383 (coin with exceptional composition), revers, area 3, SA: 150 µm × 150 µm	65.3	24.0	9.8	–	0.9	–	73.1	26.9
IAA 154383 (coin with exceptional composition), obverse, area 1, SA: 150 µm × 150 µm	77.1	14.1	8.4	0.4	–	–	84.5	15.5

IAA 154383 (coin with exceptional composition), obverse, area 2, SA: 150 μm × 150 μm	74.1	15.4	9.9	–	0.6	–	82.8	17.2
IAA 154383 (coin with exceptional composition), obverse, area 3, SA: 150 μm × 150 μm	67.3	20.5	11.1	–	1.1	–	76.7	23.3
IMJ 27383, revers, area 1, SA: 150 μm × 150 μm	95.0	–	4.4	–	0.6	–	100	–
IMJ 27383, revers, area 2, SA: 150 μm × 150 μm	96.1	–	3.9	–	–	–	100	–
IMJ 27383, revers, area 3, SA: 150 μm × 150 μm	93.9	–	5.5	–	–	0.6 Al	100	–
IMJ 27383, obverse, area 1, SA: 150 μm × 150 μm	95.3	–	4.7	–	–	–	100	–
IMJ 27383, obverse, area 2, SA: 150 μm × 150 μm	96.2	–	3.8	–	–	–	100	–
IMJ 27383, obverse, area 3, SA: 150 μm × 150 μm	93.6	–	5.5	–	0.4	0.5 Al	100	–
Trans-Jordan hoard no. 11, reverse, area 1, SA: 150 μm × 150 μm	94.1	–	5.6	–	0.3	–	100	–
Trans-Jordan hoard no. 11, reverse, area 2, SA: 150 μm × 150 μm	92.4	–	7.6	–	–	–	100	–
Trans-Jordan hoard no. 11, reverse, area 3, SA: 150 μm × 150 μm	94.0	–	6.0	–	–	–	100	–
Trans-Jordan hoard no. 11, obverse, area 1, SA: 150 μm × 150 μm	95.0	–	5.0	–	–	–	100	–
Trans-Jordan hoard no. 11, obverse, area 2, SA: 150 μm × 150 μm	91.9	–	7.7	–	0.4	–	100	–
Trans-Jordan hoard no. 11, obverse, area 3, SA: 150 μm × 150 μm	91.6	1.0	7.4	–	–	–	98.9	1.1
IMJ 34591, reverse, area 1, SA: 150 μm × 150 μm	57.1	1.8	18.3	–	15.4	7.4 Ca	96.9	3.1
IMJ 34591, reverse, area 2, SA: 150 μm × 150 μm	62.2	2.7	19.0	–	8.8	7.3 Ca	95.8	4.2
IMJ 34591, reverse, area 3, SA: 150 μm × 150 μm	61.1	1.9	15.7	–	17.0	4.3 Ca	97.0	3.0
IMJ 34591, obverse, area 1, SA: 150 μm × 150 μm	55.9	2.8	20.6	–	11.5	9.2 Ca	95.2	4.8
IMJ 34591, obverse, area 2, SA: 150 μm × 150 μm	62.7	–	16.2	–	16.2	0.6 Al, 4.3 Ca	100	–
IMJ 34591, obverse, area 3, SA: 150 μm × 150 μm	68.6	–	10.6	–	18.1	0.7 Al, 2.0 Ca	100	–

6. The Copper Concentration Distribution of Each Group of Coins

In order to determine with sufficient certainty whether our die-linked issues were produced using the same metal batch-throughout the minting processes of each series, the copper distribution of each group, based on SEM-EDS analysis, is presented (Figure S8a–h), showing the weight percentage (wt%) copper concentration range vs. the relative no. of measurements (%). For instance, 23 of the 42 (54.8%, Figure S8a) SEM-EDS measurements of the Yehud *gerah* Type 5 O1/R1 revealed composition between 0–1.0 wt% Cu, whereas all 20 Yehud quarter *obol* Type 24 O1/R2 EDS measurements revealed

composition between 0–1.0 wt% Cu (Figure S8g). The copper concentration distribution behavior of each die-linked issue shows that each series of coins was manufactured using a controlled composition of silver-copper alloy. For example, the copper concentration distribution of the Yehud *gerah* Type 5 O1/R1 (Figure S8a) is different than that of the Yehud *gerah* Type 5 O1/R2 group (Figure S8b). The copper concentration distributions of the Yehud *gerah* Type 5 O1/R3, O1/R4, O1/R5 (Figure S8c–e, respectively) are also different from the copper concentration distribution behavior of Type 5 O1/R1 and Type 5 O1/R2 coins. Moreover, the copper concentration distribution of the Yehud *gerah* Type 5 O1/R5 (Figure S8e) is more similar to that of Yehud half *gerah* Type 16 O2/R2 copper concentration distribution (Figure S8f) than to that of Yehud *gerah* Type 5 O1/R1–O1/R4 (Figure S8a–b).

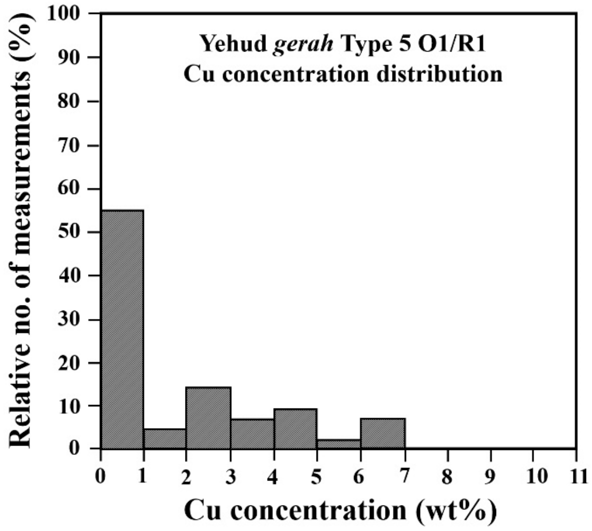

(a)

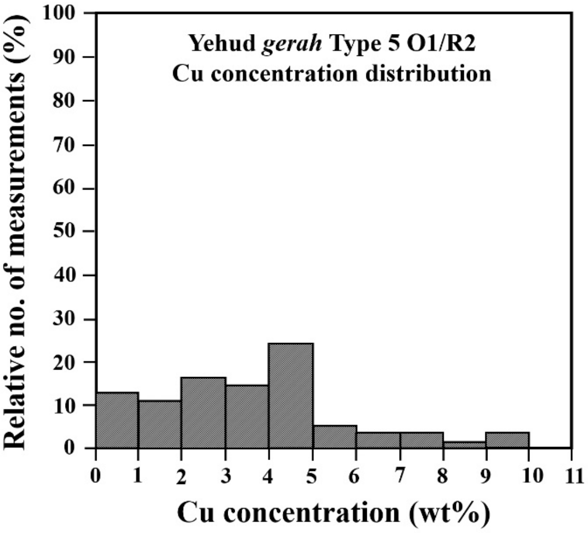

(b)

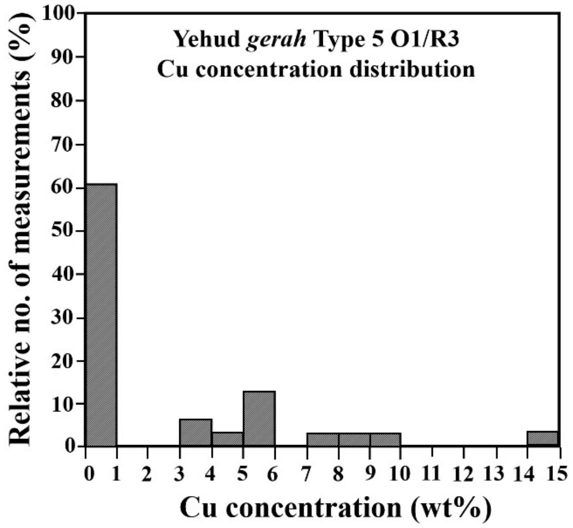

(c)

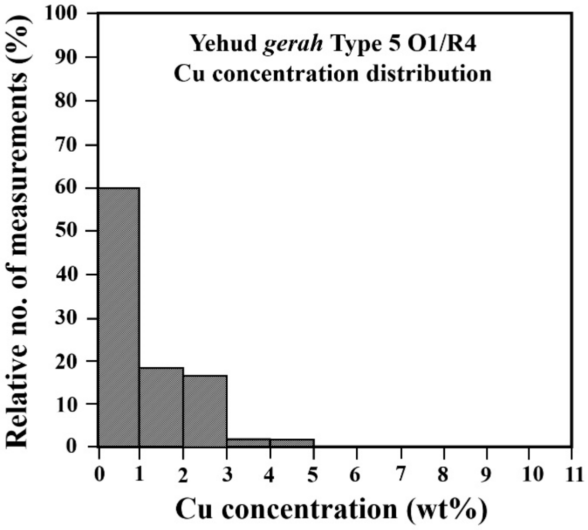

(d)

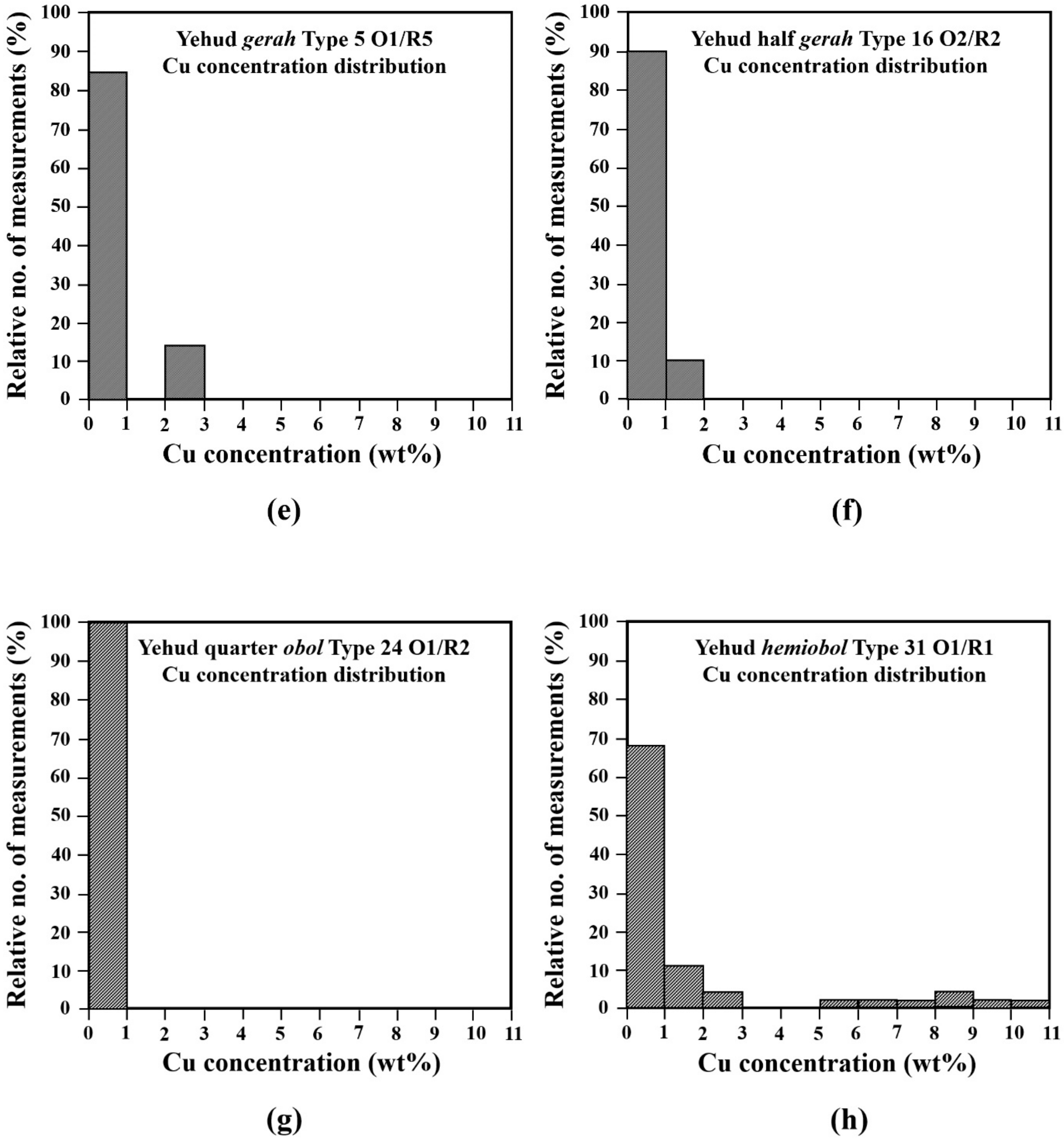

Figure S8. The copper distribution of each silver coin group based on SEM-EDS chemical analysis results, where the Cu wt% concentration is presented vs. the relative no. of measurements (%): (a) Yehud *gerah* Type 5 O1/R1; (b) Yehud *gerah* Type 5 O1/R2; (c) Yehud *gerah* Type 5 O1/R3; (d) Yehud *gerah* Type 5 O1/R4; (e) Yehud *gerah* Type 5 O1/R5; (f) Yehud half *gerah* Type 16 O2/R2; (g) Yehud quarter *obol* Type 24 O1/R2; and (h) Yehud *hemoibol* Type 31 O1/R1.

7. The Yehud Coins' Images

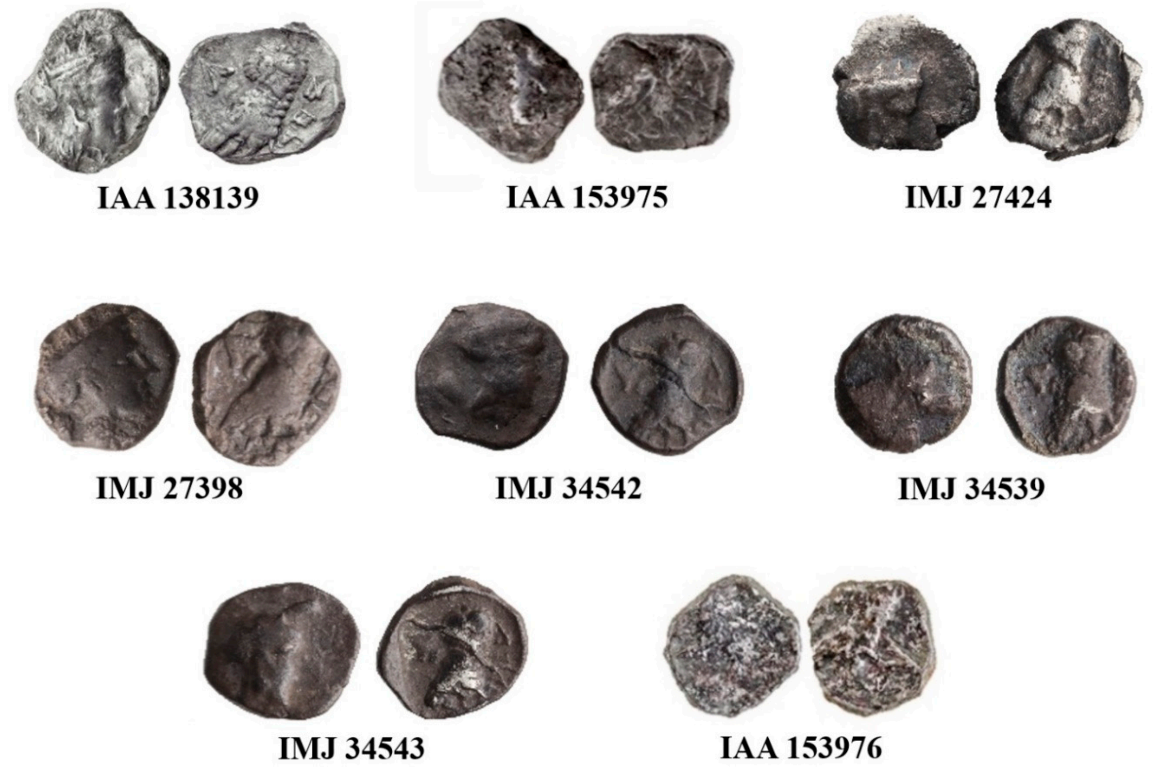

Figure S9. Yehud *gerah* coins Type 5 O1/R1 (ca. 350–333 BCE) (Athena/Owl): IAA 138139, IAA 153975, IMJ 27424, IMJ 27398, IMJ 34542, IMJ 34539, IMJ 34543, and IAA 153976.

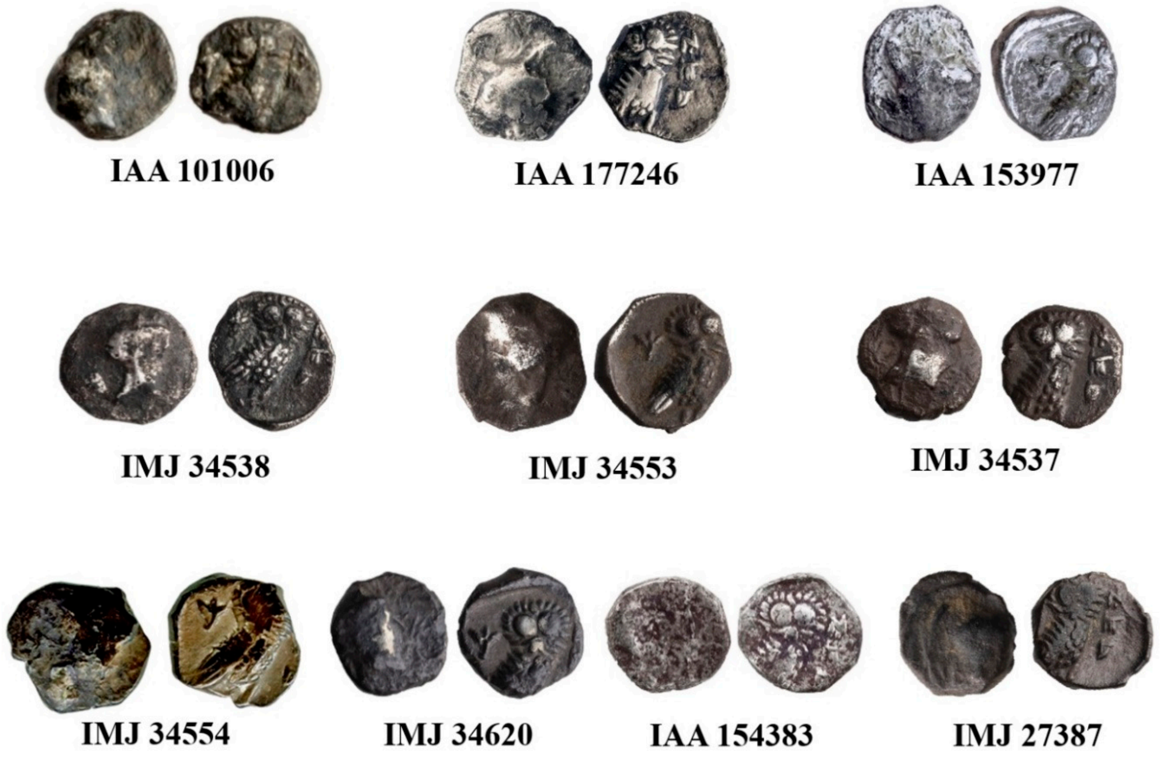

Figure S10. Yehud *gerah* coins Type 5 O1/R2 (ca. 350–333 BCE) (Athena/Owl): IAA 101006, IAA 177246, IAA 153977, IMJ 34538, IMJ 34553, IMJ 34537, IMJ 34554, IMJ 34620, IAA 154383, and IMJ 27387.

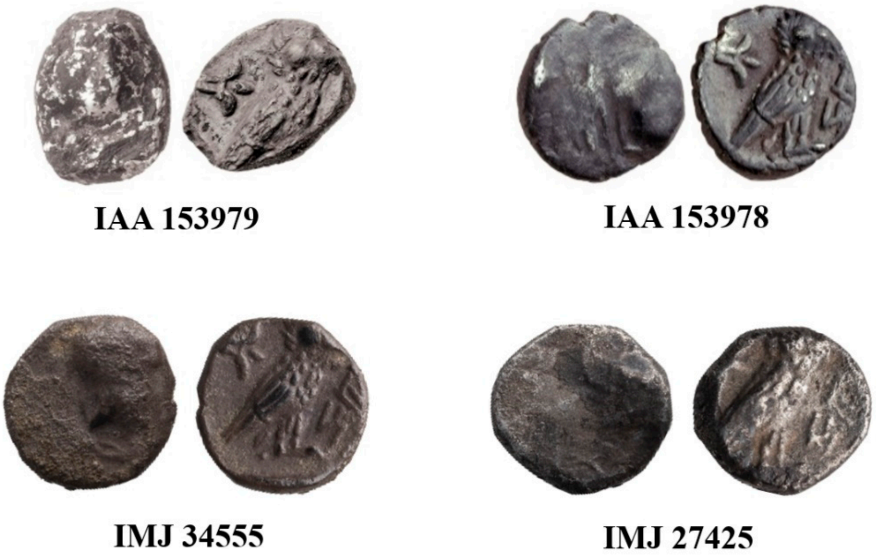

Figure S11. Yehud *gerah* coins Type 5 O1/R3 (ca. 350–333 BCE) of the Yehud series (Athena/Owl): IAA 153979, IAA 153978, IMJ 34555, and IMJ 27425.

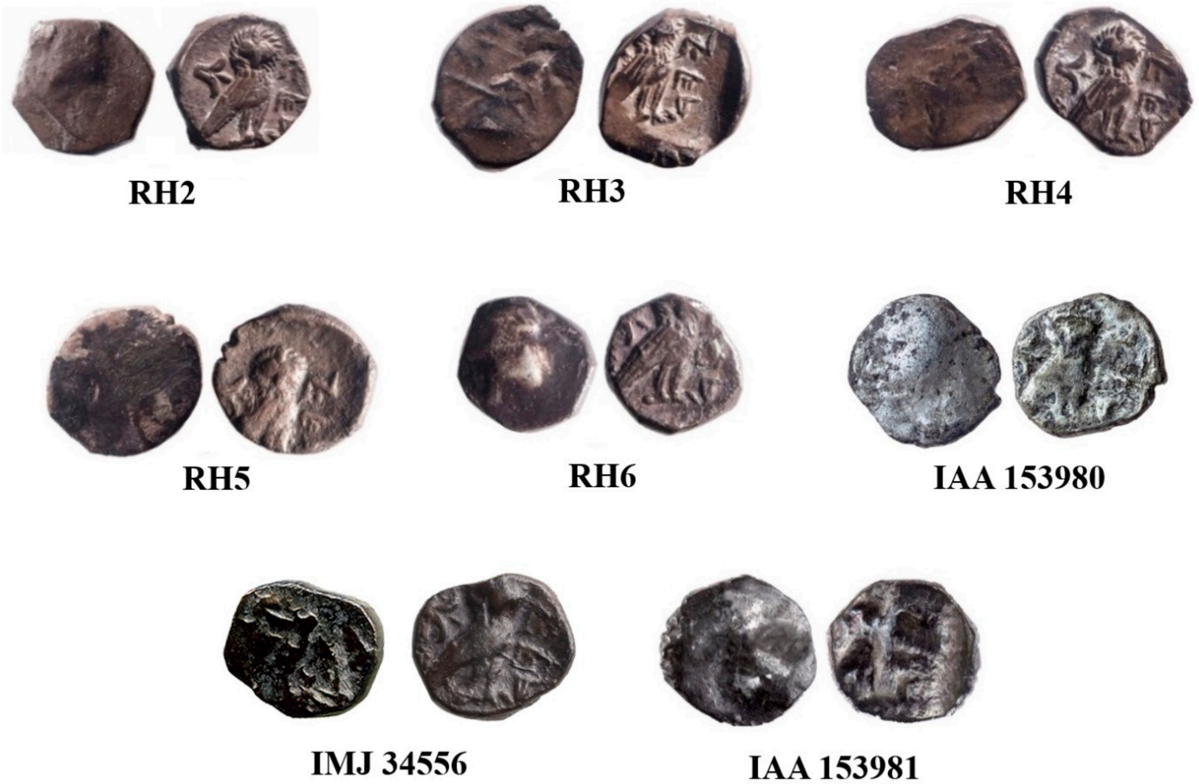

Figure S12. Yehud *gerah* coins Type 5 O1/R4 of the Yehud series (Athena/Owl): RH2-RH6, IAA 153980, IMJ 34556, and IAA 153981.

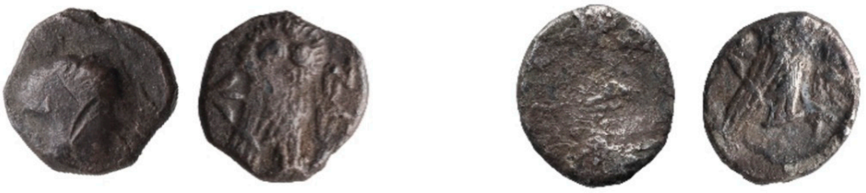

IMJ 34558 **IMJ 27388**

Figure S13. Yehud *gerah* coins Type 5 O1/R5 (ca. 350–333 BCE) of the Yehud series (Athena/Owl): IMJ 34558, and IMJ 27388.

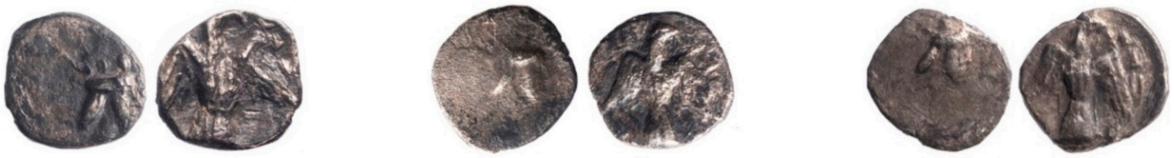

Edom 1 **Edom 2** **Edom 3**

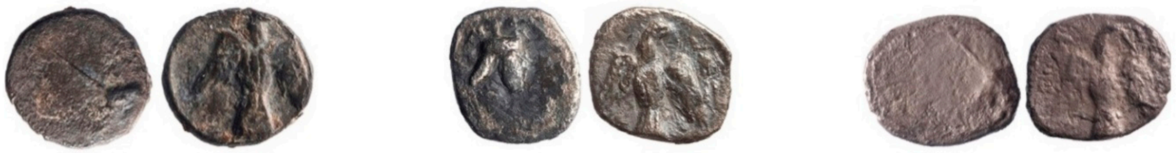

Edom 4 **Edom 5** **Edom 6**

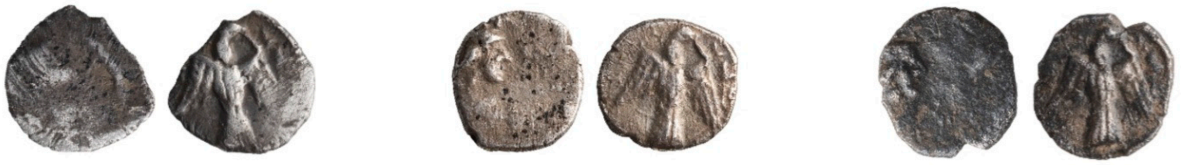

IMJ 27383 **IMJ 27414** **IMJ 34566**

Figure S14. Yehud coins Type 16 O2/R2 (Persian king wearing a jagged crown/Falcon in flight) (ca. 350–333): Edom hoard nos. 1–6, IMJ 27383, IMJ 27414, and IMJ 34566.

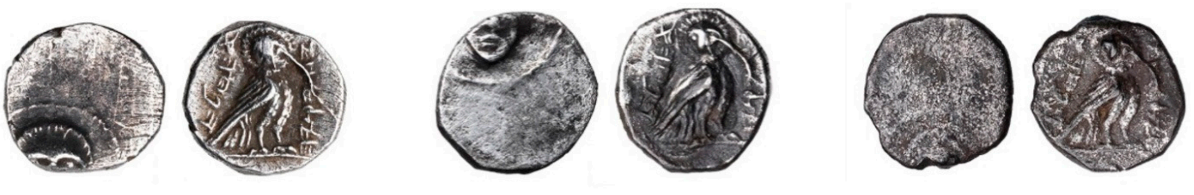

Trans-Jordan 11 **Trans-Jordan 12** **Trans-Jordan 13**

Figure S15. Yehud Attic standard quarter *obol*, Type 24 O1/R2 (Facing head/Owl) of the Macedonian period (ca. 320(?)–312 BCE): Trans-Jordan hoard nos. 11, 12, 13.

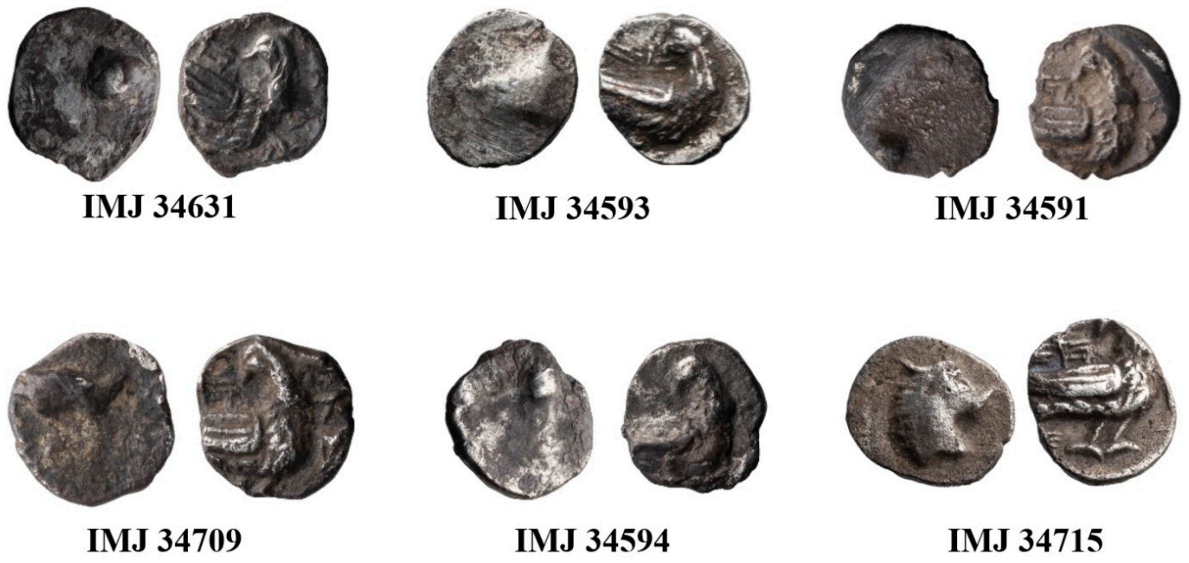

Figure S16. Yehud coins Type 31 O1/R1 (Head of roaring lion/bird standing right, head reverted) (ca. 306–302/1 BCE): IMJ 34631, IMJ 34593, IMJ 34591, IMJ 34709, IMJ 34594, and IMJ 34715.
